# Detection of low prevalence somatic mutations in solid tumors with ultra-deep targeted sequencing

**DOI:** 10.1186/gb-2011-12-12-r124

**Published:** 2011-12-20

**Authors:** Olivier Harismendy, Richard B Schwab, Lei Bao, Jeff Olson, Sophie Rozenzhak, Steve K Kotsopoulos, Stephanie Pond, Brian Crain, Mark S Chee, Karen Messer, Darren R Link, Kelly A Frazer

**Affiliations:** 1 Moores UCSD Cancer Center, University of California San Diego, 9500 Gilman Drive, La Jolla, CA 92093, USA; 2 Department of Pediatrics and Rady Children's Hospital, University of California San Diego, 9500 Gilman Drive, La Jolla, CA 92093, USA; 3 RainDance Technologies, 44 Hartwell Avenue, Lexington, MA 02421, USA; 4 Prognosys Biosciences, 505 Coast Blvd, La Jolla, CA 92037, USA; 5 Institute for Genomic Medicine, University of California San Diego, 9500 Gilman Drive, La Jolla, CA 92093, USA

## Abstract

Ultra-deep targeted sequencing (UDT-Seq) can identify subclonal somatic mutations in tumor samples. Early assays' limited breadth and depth restrict their clinical utility. Here, we target 71 kb of mutational hotspots in 42 cancer genes. We present novel methods enhancing both laboratory workflow and mutation detection. We evaluate UDT-Seq true sensitivity and specificity (> 94% and > 99%, respectively) for low prevalence mutations in a mixing experiment and demonstrate its utility using six tumor samples. With an improved performance when run on the Illumina Miseq, the UDT-Seq assay is well suited for clinical applications to guide therapy and study clonal selection in heterogeneous samples.

## Background

The number of somatic tumor mutations with potential utility for predicting treatment response is rapidly growing due to increasing numbers of targeted therapies. Clinical validation of these potential biomarkers has been slowed by both issues with tumor samples and the current paradigm underlying cancer clinical trials. Tumor DNA samples can be heterogeneous due to invasion into stroma, infiltration by immune cells, and clonal evolution. Efforts to overcome this heterogeneity have, to date, required highly focused testing of no more than a few dozen known mutations, significantly limiting progress. Additionally, cancer drug development has traditionally focused on a tissue of origin model, where efficacy studies are focused on cancers arising from one tissue type. As molecular subtyping has emerged, molecularly defined trials have been restricted to common DNA alterations (for example, Imatinib and *KIT *gene mutations in gastro-intestinal stromal tumors) [1] or uncommon alterations in very common tumors (for example, Erlotinib and *EGFR*-L858R in non-small cell lung cancer) [2]. The identification of an increasing number of somatic tumor mutations common across cancers arising from different tissues has begun to encourage molecularly defined clinical trials in which subjects with cancers from a number of differing sites of origin are eligible. To accelerate this paradigm shift, an assay capable of broad mutation testing in heterogeneous tumor samples is needed.

Somatic mutations can affect key domains of cancer genes. These mutations, associated with cancer progression and resistance to therapy, exist in restricted regions of the genome, termed mutational hotspots. Additionally, actionable mutations, in which an approved or investigational agent is available to target a pathway activated by the mutation, exist in an even more restricted set of these genomic regions. While most available clinical assays interrogate one or only a few commonly mutated loci in cancers, two published clinical assays, SNaPShot [3] and OncoMap [4], respectively target 38 mutations in 8 genes by single base extension assays and approximately 400 mutations in 33 genes by mass spectrometry. Although these assays have been extensively tested on clinical samples and are available to clinicians, they have not been thoroughly evaluated on heterogeneous tumor samples. Technological advances in DNA sequencing clearly offer an important solution to the problem of analyzing heterogeneous samples. Massively parallel sequencing enables the analysis of independent, clonal, DNA molecules [5,6] and has been used early on to digitally measure the presence of low prevalence mutations in complex DNA mixtures [7,8] or in *EGFR *exons of heterogeneous tumor DNA samples [9]. Constant improvements of the massively parallel sequencing technology offer the opportunity to revise the balance between breadth and depth of such assays and identify a wide variety of potentially actionable DNA changes in a patient's tumor. Broad assays like whole genome and whole exome sequencing have been used to discover new cancer mutations [10,11], or study clonal selection in breast cancer [12]. However, their performance on heterogeneous clinical samples has not been demonstrated and the significance of the vast majority of the mutations identified is not clear; therefore, such broad sequencing approaches currently have limited clinical utility for personalized cancer treatment. In contrast, a more targeted sequencing approach assaying all clinically actionable genes, but no extraneous regions, allows for the depth of sequencing to be maximized for a more accurate analysis of heterogeneous clinical samples.

In addition to clinical use, the efficiency of a targeted sequencing approach can also be exploited for pre-clinical drug development. The expansion and maintenance of primary tumors as xenografts in immuno-suppressed mice is commonly used in cancer research to study potential targets and evaluate therapies in a physiological context. However, the xenografting process is not neutral and can select subpopulations of cells with certain growth advantages. Enabling the comparison of the mutational profile of matched xenograft and primary tumor samples, targeted sequencing can be used to query the validity of the xenograft model.

Here, we present an Ultra-deep targeted sequencing (UDT-Seq) assay, screening 71.1 kb of sequence encompassing the mutational hotspots of 42 cancer genes. We evaluate its performance and utility by applying it to study the mutational profile of both clinical cancer and mouse xenograft samples.

## Results

### Assay design

The UDT-Seq assay is a direct sequencing method of approximately 200-nucleotide long PCR amplicons generated in multiplex using microdroplet PCR [13] (Figure S1a in Additional file 1). Briefly, we use chimeric primer pairs, containing both locus-specific and adapter sequences, to generate PCR amplicons that are then directly sequenced on the Illumina Genome Analyzer II (GAII) platform for 2 × 125-nucleotide reads. This process simplifies the workflow by removing the time consuming and error prone step of sample fragmentation and library preparation, thus providing a streamlined process for easy implementation in the laboratory. In addition, as the direct sequencing approach results in each base pair of an amplicon always being in the same position in a sequencing read (Figure S1b in Additional file 1), we are able to accurately measure the position-dependent sequencing error rate, which is known to vary significantly in sequencing by synthesis [6]. This facilitates the sensitive and specific detection of low prevalence mutations in the tumor samples. The cancer mutational hotspots screened by UDT-Seq were selected from the COSMIC database v44 [14]. An unsupervised clustering analysis (Materials and methods) led to the identification of cancer hotspots in 42 cancer genes (Table S1 in Additional file 2), which contain 53% (5,271/9,935) of all mutations and 87% (67,440/77,052) of all COSMIC database valid entries (substitutions or small indels with reported genomic location). We designed 518 primer pairs (Table S2 in Additional file 2) to amplify a total of 71.1 kb encompassing these cancer mutational hotspots.

### Assay performance

In order to estimate the error rate, train the statistical model and measure the performance of the assay, we prepared calibration samples by pooling four Coriell DNA samples (NA12156, NA12878, NA18507, and NA19240). These Coriell samples have previously been subjected to exome sequencing and thus the positions of coding polymorphisms are known [15]. We pooled these samples four times, permuting the relative concentration of the samples (1%, 5%, 20% and 74%), to obtain four different calibration samples referred to as CAL-A to CAL-D (Figure S2 in Additional file 1). The cancer hotspot amplicon library was supplemented with 158 calibration amplicons, corresponding to 23.2 kb, to detect and measure the prevalence of the alternative allele at 196 to 201 known polymorphic positions in the four CAL samples (Materials and methods). We sequenced CAL-A, CAL-B and CAL-D calibration samples once and CAL-C in duplicate, obtaining more than 30 million pairs of reads per sample, resulting in approximately 24,000-fold coverage depth after mapping (Tables S3 and S4 in Additional file 2). Consistent with our previous report [13], the coverage distribution is uniform (82% of amplicons between 0.5- and 2-fold of the mean coverage) and reproducible between samples and across sequencing runs (Figure S3 in Additional file 1 and Table S4 in Additional file 2).

We developed a four-step approach to identify low-prevalence mutations (Figure S4 in Additional file 1). In step 1 the sequencing error rate is estimated using invariant bases in a calibration sample; in step 2, the candidate mutations are filtered and their level of significance is determined in both calibration and tested samples using the error rate; in step 3, the significance threshold is calculated using the known SNPs from the calibration sample; finally, in step 4, the significant mutations are called in the tested sample using this threshold. Following this procedure we used all the CAL samples in turn for the calibration and testing, thus providing a comprehensive evaluation of the assay performance across multiple calibration-tested sample combinations and sequencing runs.

**Figure 1:**
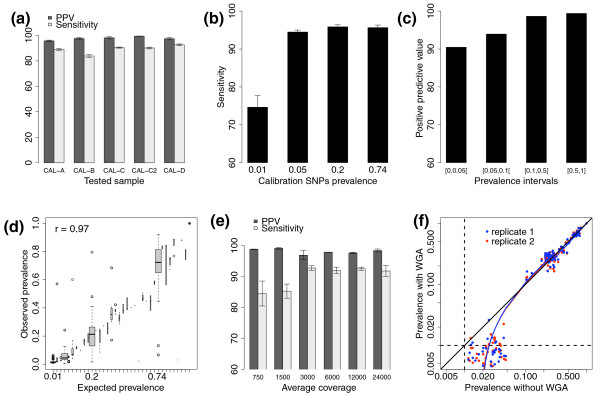
**Performance evaluation**. **(a) **The positive predictive value (PPV) and sensitivity of UDT-Seq for each of the five calibration datasets. The error bars represent the standard deviation of the values obtained from different calibration schemes (Table S5 in Additional file 2). **(b) **Average sensitivity estimation for the calibration SNPs at different prevalence's. The error bars represent the standard deviation over all calibration-tested sample combinations. **(c) **PPV at increasing prevalence intervals. **(d) **Expected prevalence of the calibration SNPs (x-axis) are highly correlated (r = 0.97) with the observed prevalence (y-axis) across all calibration samples. The width of the boxes is proportional to the root mean square of the number of SNPs in each category. The whiskers extend to the closest data point within 1.5-fold of the inter-quartile distance. On average, the mutations expected at 1%, 5%, 20% and 74% where observed at 1.5% (± 0.9), 5.4% (± 2.8), 20.3% (± 7.9) and 72.1% (± 10.6), respectively. The minor differences are likely due to measurement errors during the preparation of the calibration samples. **(e) **Average PPV and sensitivity calculated using samples CAL-C and CAL-D trained with CAL-B after random sampling the reads to lower coverage. The error bars represent the standard deviation of the results obtained from the two samples. **(f) **The prevalence of the calibration SNPs identified in CAL-B with and without whole genome amplification (WGA) (log scale x- and y-axis, respectively) is plotted against the prevalence estimated from the WGA sample replicates (red and blue). The minimum specified prevalence of the assay (1%) is indicated by dotted lines.

Out of the 23,250 bases sequenced in the calibration amplicons, we detected an average of 183 significant variants, indicating the assay specificity is greater than 99.9% (Table S5 in Additional file 2). Across all calibration-tested sample combinations, the average sensitivity was 89.1% (± 3.3%) when significant variants at 1% or greater prevalence are considered (Figure 1a). As anticipated, the sensitivity is better (> 94%) for mutations present at 5% or greater than for mutations present at 1% prevalence (75%) (Figure 1b). The average positive predictive value (PPV = 1 - (False positive rate)) is 97.6% (± 1.9%) with a noticeable lower PPV (90.5%) when expected prevalence is less than 5% (Figure 1c). As illustrated in Figure 1d, the observed prevalence is highly correlated (Pearson correlation r = 0.97) with the expected one; thus, the prevalence of the mutant allele in the DNA sample can be accurately estimated.

The initial experimental design allowed us to generate very high sequence coverage per sample (approximately 24,000-fold). A more cost-effective approach would multiplex samples using DNA barcodes, which results in a lower effective coverage. In order to estimate the effect of a reduced coverage depth on the assay performance, we sampled the reads from the full coverage from one calibration (CAL-B) and two tested (CAL-C and CAL-D) samples to simulate multiplexing levels of 2, 4, 8, 16 and 32 samples per lane corresponding to a coverage depth around 12,000×, 6,000×, 3,000×, 1,500× and 750×, respectively (Table S6 in Additional file 2). The sensitivity remains above 92% at approximately 3,000× coverage or higher (Figure 1e) but drops to 85% at 1,500× coverage depth. In contrast, the PPV remains above 96.9% at all coverage depth. Furthermore, the estimation of the prevalence remains accurate (Figure S5 in Additional file 1). Most of the false negatives were variants expected at low prevalence for which a reduced coverage leads to few reads supporting the alternative allele. Thus, these data show that the performance of the UDT-Seq assay is maintained at an average coverage of approximately 3,000× and greater.

### Identification of somatic mutations in cancer samples

We sequenced 71.1 kb of cancer mutational hotspots in DNA samples from a primary colon adenocarcinoma with its matching xenograft, a breast primary carcinoma with its matching xenograft, an ovarian carcinoma xenograft, a sarcoma xenograft (Materials and methods) and the matching germline DNA derived from all four patients' blood. The xenograft samples had been passaged between two and seven times in immunodefficient mice (Materials and methods). To call the significant mutations, we trained the method with a calibration sample sequenced in the same run as the clinical sample (Table S7 in Additional file 2). As shown above, the performance of UDT-Seq is significantly better for higher prevalence mutations. For this reason, we restricted our analysis to the mutations identified at a prevalence of 5% or greater. The somatic mutations were then called by analyzing the differences between cancer and germline DNA mutations. We considered all variants in a cancer sample that passed statistical assessment (Materials and methods) as potential somatic mutations. We then retained the mutations for which the corresponding position in the germline sample was covered (> 10 reads) and is either 1) identified as not variant by the statistical analysis or 2) shows little evidence of the alternative allele (< 20% alternative allelic ratio). Across the six samples analyzed (2 primary and 4 xenografts samples), we discovered 13 unique somatic mutations. To understand UDT-Seq assay performance, we first examined all mutations by visual inspection of the reads and then independently validated a subset using sequence-based assays (SNaPshot or Sanger).

**Figure 2:**
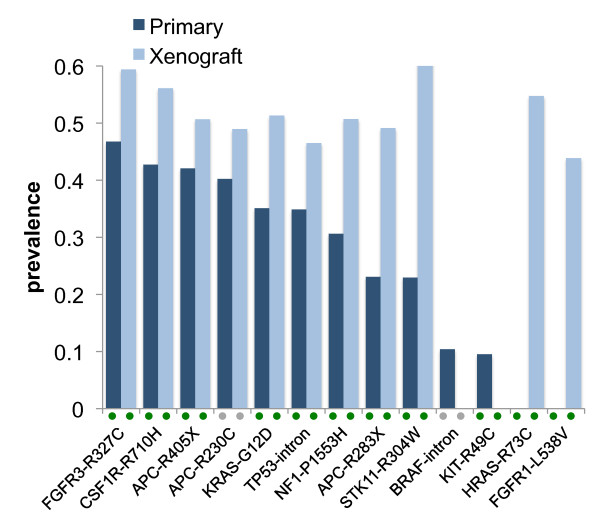
**Mutational profiles of the colon primary and xenograft tumor samples**. Histogram showing the prevalence of the 11 colon primary (dark blue) and 11 xenograft (light blue) mutations. In the primary tumor, ten of the mutations were identified using the UDT-Seq method and one (STK11-R304W) by visual inspection of the reads after it was observed in the xenograft. All mutations validated by a sequence-based assay (SNaPshot or Sanger) are indicated by a green dot. Mutations not examined by independent assay are indicated by a grey dot.

In the colon primary sample, UDT-Seq identified ten somatic mutations (Figure 2, Table 1; Table S8 in Additional file 2) of which eight are shared with the xenograft sample and two are present only in the tumor. Seven of these mutations are possibly heterozygous in a majority of the cells (prevalence between 31% and 47%) and three have an intermediate prevalence (10% to 23%). This distribution suggests the presence of different cell populations. Of note, KRAS-G12D has a high prevalence (35%); this cancer driver mutation is present in 40% of colorectal cancers and is associated with anti-EGFR therapy resistance [16]. Interestingly, two common *APC *inactivating mutations (APC-R405X and APC-R283X), both frequent mutations in colorectal cancer, are present at different prevalence (25% and 49%, respectively), suggesting that they occur in different cell populations. Examination of the primary specific mutations (BRAF-intron and KIT-R49C) shows that neither of them has evidence of presence in the xenograft (Figure 2). In the matching colon xenograft sample, we identified 11 somatic mutations, of which 8 are shared with the primary (Figure 2, Table 1). Examination of the three xenograft-specific mutations revealed that two were well covered in the primary without evidence of the mutant allele, but one (STK11-R304W) was a false negative in the primary (filtered due to relatively low coverage) and validated by an independent assay at 23% prevalence (Table S9 in Additional file 2). Interestingly, this mutation has been identified in patients with Peutz-Jeghers syndrome, an inherited cancer syndrome associated with intestinal polyps and cancer risk [17]. Both STK11-R304W and APC-R283X show significant and similar prevalence differences in the primary (22 to 23%) and xenograft (49%), suggesting that they may be present in the same populations of cells, and were selected for in the xenograft.

**Table 1:** Prevalence of all somatic mutations identified by UDT-Seq using a 5% prevalence detection threshold

Cancer		Validation	Prevalence estimated by UDT-Seq		
			
type	Mutation	assay	Primary	Xenograft	Xenograft + WGA
Colon	FGFR3-R327C	Sanger	**0.47**	**0.59**	
	CSF1R-R710H	SNaPshot	**0.43**	**0.56**	
	APC-R405X	SNaPshot	**0.42**	**0.51**	
	APC-R230C	Not assayed	0.4	0.49	
	KRAS-G12D	SNaPshot	**0.35**	**0.51**	
	TP53-intron	SNaPshot	**0.35**	**0.47**	
	NF1-P1553H	SNaPshot	**0.32**	**0.51**	
	APC-R283X	SNaPshot	**0.23**	**0.49**	
	BRAF-intron	Not assayed	0.1	< 0.05	
	KIT-R49C	Sanger	**0.1**	**< 0.05**	
	STK11-R304W	SNaPshot	< 0.05*	**0.66**	
	HRAS-R73C	SNaPshot	**< 0.05**	**0.55**	
	FGFR1-L457V	SNaPshot	**< 0.05**	**0.44**	
Breast	HRAS-G12V	Not assayed	0.51	0.48	0.48
Ovarian	TP53-R248Q	Not assayed		1	

*Inspection showed it to be a false negative (present by visual inspection). Independently validated mutations (or absence thereof) are indicated in bold. WGA, whole genome amplification.

In the primary breast cancer sample, we identified a single somatic mutation (HRAS-G12V) by UDT-Seq (Table 1; Table S8 in Additional file 2). HRAS-G12V (prevalence 51%) is a common activating mutation in bladder cancer [18], and its role in breast cancer has not been described. HRAS-G12V is homologous to the KRAS-G12D mutation and similarly may be important in the development of resistance to tyrosine kinase targeting agents [16]. This mutation was also identified in the matching xenograft sample at a similar prevalence (48%), suggesting its importance for breast cancer growth and proliferation.

Overall our comparison between primary and xenograft samples reveals evidence of tumor heterogeneity and the presence of sub-clonal cell populations. The mutations identified are mostly non-synonymous and some can have direct clinical impact as they are markers of poor prognosis or predictive of drug resistance. Interestingly, the colon primary sample presented the most complex and rich mutational profile, which, with the exception of a few mutations, was faithfully matched in a xenograft after seven successive passages in mouse. While other possible somatic changes, such as copy number alterations, were not assessed, this observation supports the use of xenograft models to reflect the genetics of the primary tumor, in agreement with previous studies [12].

To complete our assessment of tumor heterogeneity via UDT-Seq, we further analyzed the sequences derived from the ovarian adenocarcinoma and small intestine sarcoma xenografts and their matched germline DNA. The ovarian xenograft shows a homozygous somatic mutation at TP53-R248Q, the most common inactivating mutation in *TP53 *(Table 1), a gene mutated in 96% of ovarian cancers [19]. We did not identify any mutations in the sarcoma xenograft sample. These additional results confirm that UDT-Seq can identify known and novel mutations in previously uncharacterized samples.

### Effect of whole genome amplification

Cancer samples, and biopsy in particular, can generate low amounts of total DNA. Whole genome amplification (WGA) by multiple strand displacement is a popular method to increase the amount of material available for clinical assays [20]. We evaluated the effect of WGA on the CAL-B calibration sample by comparison with the non-amplified sample. Both sensitivity and PPV were unchanged (Figure S7 in Additional file 1). Surprisingly, in the WGA-amplified samples the observed prevalence of mutations expected at 5% or less dropped significantly (Figure 1f). This is likely due to allele-specific bias generated during the amplification. We then applied WGA to the breast cancer xenograft sample and performed a UDT-Seq assay, observing the HRAS-G12V mutation at 49%, in agreement with the prevalence observed without WGA (Table 1). Because the initial sample did not carry any low prevalence mutations, we could not verify the potential allelic bias below 5%. Thus, UDT-Seq analysis of DNA samples subjected to WGA provides reliable results for highly prevalent mutations but underestimates the presence of low prevalence alleles.

### Implementation of UDT-Seq on a MiSeq sequencer

Fast turn-around time of an assay like UDT-Seq is important for its clinical implementation. The sequencing presented above requires approximately 12 days of an Illumina GAII run to complete. Recent technology developments have resulted in the commercialization of new, smaller instruments that are time- and cost-effective while still providing a sufficient yield compatible with UDT-Seq breadth and depth. Using multiplexing adapters, we sequenced the same four calibration samples (CAL-A to CAL-D) in one run of Illumina MiSeq (Materials and methods). This resulted in an average depth of 1,571× per amplicon. Using an analysis strategy strictly identical to the one described for the GAII data, we noticed a significant reduction of the substitution rate, especially at the end of the reads for 'A' and 'T' reference bases (Figure S8a-d in Additional file 1). These improvements are the consequence of a better chemistry since the initial GAII run as well as faster cycling time. This resulted in a more than six-fold reduction in the number of positions determined as significantly noisy by filter 7 (Figure S8e in Additional file 1). As a result, the sensitivity of the assay improved from 85% (Figure 1e) to approximately 90% (Figure S8f in Additional file 1) when comparing the GAII and MiSeq data at equivalent coverage depth. The PPV remains very high, witnessing to the robustness of the initial statistical analysis strategy.

## Conclusions

The prevalence of the mutations detected in complex DNA mixture has traditionally been limited to approximately 20% using Sanger sequencing [21,22]. The development of specific mutation enrichment or detection strategies has greatly increased this sensitivity [3,23,24], but impaired the breadth of the assay. The UDT-Seq approach presented here offers a streamlined method to implement in clinical care massively parallel sequencing of cancer mutational hotspots in heterogeneous samples. The simultaneous sequencing of a calibration sample enhances the robustness of the assay and therefore the reliability of the results. We have shown that this approach can comprehensively detect low prevalence mutations by screening 71,081 DNA positions located in cancer mutational hotspots. The sensitivity of the assay down to mutations present at 5% prevalence permits detection of mutations in heterogeneous or poor quality samples with rare mutated clones, low cellularity, or contamination with stroma or immune cell infiltration, all of which are commonly seen in clinical samples. Importantly, our data suggest that in order to increase the reliability and identify mutations present at less than 5% prevalence, the accuracy of the next generation sequencing technology needs to increase, with improvements of both chemistry, instrument and bioinformatics analysis. Increasing sequence depth coverage only is unlikely to solve the systematic bias observed that limits the ability to accurately measure the abundance of alleles present at less than 5% prevalence. This is exemplified by the notable improvement in the substitution rate observed on the MiSeq instrument, where the samples were sequenced at lower depth.

The UDT-Seq assay will enable high throughput molecular testing for a large number of cancer patients that have samples that are incompatible with current comprehensive diagnostic procedures. By integrating this tool in institutional master clinical protocols, it can immediately enable focused clinical confirmatory sequencing for selection of patients for targeted treatments or clinical trials testing novel targeted therapies or repurposing of approved drugs. Going forward we expect that this tool will be deployed in clinical testing, further facilitating its use for clinical management of patients. Additionally, UDT-Seq will empower the study of clonal selection in cancer metastasis, recurrence and progression. Comparison of initial UDT-Seq profiles with disease outcomes may identify novel targets that, with therapeutic intervention, can prolong survival or reduce mortality. Lastly, similar to other approaches [25], UDT-Seq can also be used to establish a personalized molecular signature of tumor driver and/or passenger mutations that can be used to monitor for recurrence or response in circulating DNA in plasma or urine by more sensitive methods.

## Materials and methods

### Assay overview

The UDT-Seq is a direct sequencing method of approximately 200-nucleotide-long PCR amplicons generated in multiplex using microdroplet PCR [13] (Figure S1a in Additional file 1). Briefly, we use chimeric primer pairs containing both locus-specific and partial Illumina adapter sequences to generate PCR amplicons in droplets, followed by the breaking of the emulsion and a secondary universal PCR amplification with primers that incorporate the remainder of the Illumina adaptor sequences. These amplicons are then directly sequenced on the Illumina platform for 2 × 125-nucleotide reads. This process removes the time consuming and error prone steps of sample fragmentation and library preparation, thus providing a streamlined process for easy implementation in the laboratory. In addition, as the direct sequencing approach (Figure S1b in Additional file 1) results in each base pair of an amplicon always being in the same position in a sequencing read, we are able to accurately estimate the position-dependent sequence read error rate, which is known to be variable in sequencing by synthesis [6]. This facilitates the sensitive and specific detection of low prevalence mutations in the tumor samples. We designed 676 primer pairs to target 518 cancer mutational hotspots of 42 cancer genes, as well as 158 calibration amplicons (see below; Table S2 in Additional file 2).

### Cancer hotspot selection

The cancer mutational hotspots were selected from the COSMIC database v44 [14]. We initially selected all non-synonymous mutations involving single base substitutions and indels shorter than 140 bp in length. These criteria identified 9,935 mutations affecting 2,468 genes. The COSMIC database has some redundancy due to over-ascertainment of particular genes or cancer types; 96% of the mutations were observed less than 5 times and 2% were observed more than 100 times. Based on this clustering analysis, we chose 42 cancer genes (Table S1 in Additional file 2), which contain 53% (5271/9935) of all mutations and 87% (67,440/77,052) of all COSMIC (v44) valid entries (substitutions or small indels with reported genomic location). Only 141 of the 5,271 mutations were singletons defined as located more than 140 bp from another mutation (maximum length that can be assayed in one amplicon). We designed 518 primer pairs to amplify a total of 71 kb encompassing the 5,271 mutations.

### Calibration samples and SNP selection

In order to estimate the error rate, train the statistical model and measure the performance of the assay, we prepared calibration samples by pooling four Coriell DNA samples (NA12156, NA12878, NA18507, or NA19240). These Coriell samples have previously been subjected to exome sequencing and thus the positions of coding polymorphisms are known [15]. We pooled these samples four times, permuting the relative concentration of the samples (1%, 5%, 20% and 74%), to obtain four different calibration samples referred to as CAL-A to CAL-D. We selected 40 calibration coding SNPs based on the fact that they were homozygous for the alternate allele in one of the four samples and homozygous for the reference allele in the three others. We repeated this selection four times, permuting the four samples, resulting in a total of 160 calibration SNPs. We successfully designed primer pairs to amplify 200-bp fragments around 159 of these SNPs. Because we perform direct sequencing from the amplicon ends (Figure S1b in Additional file 1), we made sure that the calibration SNPs were located at various positions throughout the 200-bp amplicons. This allowed us to estimate the assay performance for calling variants across the 200-bp amplicons. Of the 159 primer pairs tested, 158 successfully amplified the targeted sequences in the four calibration samples. Other SNPs, not initially selected to serve as calibration polymorphisms, are located in these 158 amplicons. We disregarded heterozygous SNPs present only in one of the four Coriell samples, as they would lead to a prevalence of 0.5% in one of the calibration samples. In total this design resulted in 196, 200, 201 and 201 SNPs with known prevalence in the calibration samples CAL-A, CAL-B, CAL-C and CAL-D, respectively. The UDT-Seq assay interrogates a total of 23.2 kb encompassing these calibration SNPs.

### Primer library design

The custom primer library was designed using the Primer3 algorithm and the manufacturer's suggested parameters (RainDance Technologies Lexington MA, USA). The locations of SNPs in dbSNP build 128 were masked and not used as potential sites for primer selection. Repeat masking was not performed on the input sequences to the primer design pipeline. The primer design pipeline performed exhaustive primer selection across the targeted intervals. Targeted regions that failed to produce PCR primers with the standard parameters through the automated pipeline were designed manually, altering different standard parameters until a successful design was achieved (Table S2 in Additional file 2). After designing the locus-specific portion of the primers, sequence tails corresponding to a portion of the Illumina adaptor sequence were added to the sequence-specific portion of the primers prior to synthesis. The sequence added to the forward primers was CGCTCTTCCGATCT**CTG **and the sequence added to the reverse primer was CGCTCTTCCGATCT**GAC**. The tri-nucleotide sequence in bold is inserted between the target specific and the universal primer to confer adapter-strand specificity so that only the reads originating from the same end of the amplicon will be sequenced simultaneously (Figure S1b in Additional file 1). This ensures that the sequencing error rate can be computed as a function of the read direction (forward or reverse).

### Sample preparation

#### Calibration samples

The four Coriell Institute DNA samples (NA12156, NA12878, NA18507, and NA19240) were quantified in triplicate using Nanodrop and each sample was diluted at a low concentration (approximately 5 ng/μl), which was re-measured in triplicate by Nanodrop (Agilent Technologies Santa Clara CA, USA). Based on these measurements, the four calibrations samples were prepared by mixing the appropriate volume of the initial sample (for the 74% and 20% prevalence) or of the diluted samples (for the 5% and 1% prevalence) for a total of 7.5 μg of DNA per calibration sample (Figure S2 in Additional file 1).

#### Cancer specimen description

Human primary tumors and whole blood were collected under the UC San Diego IRB-approved protocol with prospective consent. The participants were enrolled in accordance with the Helsinki Declaration and to local legislation and gave informed consent. The breast cancer corresponds to a primary metaplasmic carcinoma from a 62-year-old patient (T2, N0, M0) untreated at sampling. The sample was negative for progesterone receptor, HER2 and focally positive (< 5%) for estrogen receptor as estimated by immunohistochemistry. The colon cancer corresponds to an enteric adenocarcinoma from a 65-year-old patient (T3, N0, M0) untreated at sampling. The sample was positive for MLH1, PMS2 and CD20, and in a few cells for MSH2, and negative for MSH6 by immunohistochemistry. The ovarian cancer corresponds to a serous adenocarcinoma of enteric type from a 64-year-old patient (T3b, N0, M0) treated with three cycles of idarubacin and paclitaxol before sampling. The sarcoma sample corresponds to a small intestine pleomorphic sarcoma identified in a 59-year-old patient (metastasis M1) from recurrent disease following chemotherapy and 3 years after initial diagnosis.

#### Specimen preparation

The buffy coats were prepared from whole blood and resuspended in RNAlater (Applied Biosystems, Foster City, CA, USA). Tumor tissue was minced using an autoclaved razor blade to create a slurry and mixed with an equal volume of high concentration matrigel (BD Biosciences, San Jose, CA, USA). Tumors were implanted subcutaneously in NSG mice (exact strain name NOD.Cg-*Prkdc^scid ^Il2rg^tm1Wjl^*/SzJ; The Jackson Laboratory, Sacramento, CA, USA). Representative portions of each passage were re-implanted, formalin fixed or snap-frozen in liquid nitrogen for archival use. The breast, colon, ovarian and sarcoma xenografts were passaged in mice 2, 7, 5 and 2 times, respectively, before sampling for our study. Snap-frozen tissue samples were subjected to mechanical pulverization, followed by disruption of the tissue in lysis buffer and DNA/RNA extraction using AllPrep DNA extraction kits (Qiagen GmbH Hilden Germany).

### Whole genome amplification

Purified genomic DNA (100 ng) was amplified using REPLI-g Mini WGA Kit (Qiagen) following the manufacturer's instructions. The amplified DNA was purified and quantitated by UV spectrometry (Nanodrop).

### Microdroplet PCR

#### Genomic DNA preparation

Genomic DNA samples were fragmented using a nebulization kit (Invitrogen, K7025-05 Carlsbad, CA, USA) following the manufacturer's recommended protocol: 2.5 μg of genomic DNA was re-suspended in 750 μl Shearing Buffer (TE, pH 8.0 (Fisher Waltham MA, USA, 50843207) containing 10% glycerol (Fisher, AC15892)) and was nebulized at 6 to 10 pounds per square inch (psi) for 90 seconds to produce 2- to 4-kb DNA fragments. Fragmentation of the genomic DNA to 2 to 4 kb allows for optimal template size for performing PCR in droplets. Sheared genomic DNA was precipitated by adding 80 μl 3 M sodium acetate, pH 5.2 (Fisher, 50843081), 4 μl 20 mg/ml Mussel Glycogen (Fisher, NC9329100) and 700 μl 100% isopropanol (Fisher, AC14932). Samples were mixed by inversion and stored overnight at -20°C. The samples were centrifuged at the maximum speed (21,000 g) for 15 minutes at 4°C. The supernatant was discarded, 500 μl of cold 80% ethanol (Fisher, 5739852) wash buffer was added and the DNA pellet was spun down by centrifugation at the maximum speed for 5 minutes at 4°C. The pellet was air dried at room temperature for 20 minutes and re-suspended in 10 μl 10 mM Tris-HCL, pH 8.0 (Sigma St Louis MO, USA, T2694). Fragmented genomic DNA was analyzed by gel electrophoresis on a 0.8% agarose gel to confirm that the genomic DNA was in the correct size range (2 to 4 kb). To prepare the input DNA template mixture for targeted amplification, 1.5 μg of the purified genomic DNA fragmentation reaction was added to 4.7 μl 10× High-Fidelity Buffer (Invitrogen, 11304-029), 1.26 μl of MgSO_4 _(Invitrogen, 11304-029), 1.71 μl 10 mM dNTP (New England Biolabs, Ipswich MA, USA, NO447S/L), 3.6 μl Betaine (Sigma, B2629-50G), 3.6 μl of RDT Droplet Stabilizer (RainDance Technologies, 30-00826), 1.8 μl dimethyl sulfoxide (Sigma, D8418-50 ml) and 0.72 μl 5 units/μl of Platinum High-Fidelity Taq (Invitrogen, 11304-029) and the samples were brought to a final volume of 25 μl with nuclease free water (Teknova-Fisher Hollister CA, USA, 50843418).

#### Droplet merge on the RDT1000

PCR droplets were generated on the RDT1000 (RainDance Technologies, 20-01000) using the manufacturer's recommended protocol: 25 μl of the DNA template mixture in a tube, the custom primer droplet library in a separate tube and a disposable microfluidic chip (RainDance Technologies) were placed onto the RDT1000. The custom primer droplet library consisted of the primer pairs described in the 'Library primer design' section above where each primer droplet contained matched forward and reverse PCR primer pairs (5.2 μM per primer). The RDT1000 generates each PCR droplet by merging a single DNA template droplet with a single primer droplet [13]. Each PCR droplet contains a final primer concentration of 1.6 μM per primer. The PCR droplets are automatically dispensed as an emulsion into a PCR tube per each test sample and transferred to a standard thermal cycler for PCR amplification. Each single sample generated more than 1,000,000 single plex PCR droplets.

#### Microdroplet PCR

Samples were cycled in a Bio-Rad (Hercules CA, USA) PTC-225 thermal cycler as follows: initial denaturation at 94°C for 2 minutes; 55 cycles at 94°C for 15 seconds, 58°C for 15 seconds and 68°C for 30 seconds; final extension at 68°C for 10 minutes, followed by a 4°C hold. Following PCR amplification, the emulsion of PCR droplets was broken to release each individual amplicon from the PCR droplets. For each sample, an equal volume of RDT 1000 Droplet Destabilizer (RainDance Technologies, 40-00830) was added to the emulsion of PCR droplets, the sample was vortexed for 15 seconds and then centrifuged at 12,000 × g for 10 minutes. The oil from underneath the aqueous phase was carefully removed from the sample. Each sample was purified over a MinElute column (Qiagen, 28004) following the manufacturer's recommended protocol. The sample was eluted off the column with 11 μl of the Qiagen Elution Buffer. Purified amplicon DNA was then analyzed on an Agilent Bioanalyzer to quantify amplicon yield.

#### Universal PCR

Four microliters of amplicons (2.5 ng/μl), as determined by Agilent BioAnalyzer quantification from the initial droplet PCR, were combined with 2.5 μl 10× High-Fidelity Buffer (Invitrogen, 11304-029), 1.0 μl of MgSO_4 _50 mM (Invitrogen, 11304-029), 1.13 μl of 10 mM dNTP (New England Biolabs, NO447S/L), 2.5 μl of 4 M Betaine (Sigma, B2629-50G), 2.5 μl of RDT Droplet Stabilizer (RainDance Technologies, 30-00826), 1.25 μl dimethyl sulfoxide (Sigma, D8418-50 ml), 5.0 μl of a 0.5 μl 5 units/μl of Platinum High-Fidelity Taq (Invitrogen, 11304-029), 4.62 μl of Nuclease Free Water (Teknova-Fisher, 50843418) and 1 μM final of each universal primer, incorporating the remaining sequence to the Illumina adapter for cluster generation and sequencing. Samples were amplified in a Bio-Rad PTC-225 thermal cycler as follows: initial denaturation at 94°C for 2 minutes; 8 cycles at 94°C for 15 seconds, 56°C for 15 seconds and 68°C for 30 seconds; final extension at 68°C for 10 minutes, followed by a 4°C hold. Each sample was purified over a MinElute column (Qiagen, 28004) following the manufacturer's recommended protocol. The sample was eluted from the column with 11 μl of the Qiagen EB buffer. The purified amplicon DNA was then analyzed on an Agilent Bioanalyzer to quantify final amplicon yield.

### Experimental design

The global experimental design is described in Figure S9 in Additional file 1. In the first stage of the study, we used the four CAL samples to estimate the performance of the assay. The performance was evaluated by successively using one CAL sample to calibrate the algorithm and a different one as a test sample, performing all possible permutations between the four CAL samples as calibration and test samples (Table S5 in Additional file 2). The second stage of the study applied the algorithm to cancer samples (primary and xenografts) using the CAL samples sequenced on the same run for calibration.

### Primary data analysis

Reads were aligned to the hg19 genome using BWA (version 0.5.7) [26] in single end mode (samse option). Using SAMTOOLs [27], the reads containing gapped alignments ([ID] MD tag) were further filtered out. Our approach, relying on error rate estimation and statistical analysis was optimized to identify nucleotide substitutions. The detection of indels from gapped alignment requires a more sophisticated approach, which will be developed in future studies. Reads coming from the forward and reverse strands were kept separate for the analysis, to estimate the sequencing error rate independently for each read direction. In addition, for positions contained on two types of reads (either forward and reverse or from overlapping amplicons) an independent statistical analysis was performed. Each read was assigned to one amplicon based on its start coordinate. For each base of each amplicon, we counted the number of each nucleotide with PHRED quality greater than 20 to generate a 'pileup' table. The files corresponding to the raw reads are publicly available on the NCBI Short Read Archive (SRP009487.1) [28].

### Coverage calculations

Base-wise coverage depth calculations (Table S4a in Additional file 2) were performed using collapsed alignment (forward and reverse reads merged). Amplicon-wise coverage depth calculations (Table S4b in Additional file 2) were performed using reads aligning to each specific amplicon (based on start site coordinate). This way, amplicons that overlap are kept separate for the coverage calculation and do not artificially inflate the coverage of the overlapping bases.

### Mutation detection procedure

The procedure described below combines four preliminary filters, the statistical assessment and three post-analysis filters to optimize the use of sequencing information and the computational efficiency.

#### Step 1: error rate estimation

Using the 94.3 kb (23.2 calibration + 71.1 kb mutation hotspots) of DNA sequenced for assay calibration, there were 2,770 SNP positions in only one of the four CAL samples recorded in the dbSNP131 database. We first masked these positions examining only invariant bases to estimate the position-dependent error rate. The error rate is defined, at each invariant base, as the fraction of non-reference bases. Any position with an alternative frequency greater than 0.05 was filtered out because this would most likely result from a non-annotated SNP or a PCR error occurring upstream of sequencing. After filtering out the positions covered by less than ten reads, we calculated the error rate as a function of the strand (forward or reverse), the position on the reads (1 to 122) as well as the type of substitution observed (4 × 3 = 12 possibilities when considering strands separately), which are usual predictors of error in sequencing by synthesis [6]. The retained positions were stratified into bins corresponding to the substitution type (reference to mismatch substitution), the read position, and the strand. An error rate was estimated by grouping all the observations from a specified bin. Grouping is critical given that the variation in read depth is variable among the observations. In order to correct for the noise from the low number of observations in some cases, the error rate was smoothed borrowing information from nearby read positions to decrease the noise in the error rate estimation. Smoothing was accomplished within each bin stratified by the reference allele, the alternative allele and the strand. Given a read position k, if the sum of read depth is less than the average depth across all bins, then the estimated error rates from read positions of k+1, k-1, k+2, k-2, and so on, were used to smooth the estimation until the sum of read depth exceeded the average depth across all bins. The contributions were weighted by the distance to k as well as by the read depth. The smoothed error rate was computed as E = e × n × r, where e is the error rate, r is the reciprocal of the distance to read position being smoothed (for example, 1/2, 1/3) and n is the read depth. The smoothed error rate as a function of read direction, read position and substitution type is presented in Figure S8 in Additional file 1.

#### Step 2a: filtering of candidate mutations

We first applied some general filtering rules to the dataset to improve the computation time. Filtering out a position equates to calling it a non-mutated site. The filters used for analysis can be divided into four sequential rules.

##### Filter 1

Positions located within primer sequences are filtered out.

##### Filter 2

Any position with a fraction of non-reference alleles less than 0.2% of the total coverage at this position is filtered out. This threshold was used based on the initial specification of UDT-Seq to identify variants present at 1% prevalence. An alternative allele with five times lower support than 1% prevalence is unlikely to be a true positive.

##### Filter 3

For positions covered by a single read (forward or reverse), we reject all positions located more than 100 nucleotides from the read start, to prevent increasing error rate at the end of the reads.

##### Filter 4

Reads (122 nucleotides) are sequenced from both ends of the amplicons (approximately 200 bp long). Therefore, some positions are sequenced from both reads (R and F for reverse and forward), which sometimes have inconsistent calls. At these positions we reject the F read call (respectively R read call) if 1) *Cov(F) < 0.1 Cov(R) *(respectively *Cov(R) < 0.1 Cov(F)*), where *Cov *indicates coverage depth at the examined position, or if 2) *Pos(R) > Pos(F) *(respectively *Pos(F) > Pos(R)*), where *Pos *indicates the distance in nucleotides from the read start.

#### Step 2b: determination of the statistical significance

Given the estimated error rate E, the read depth N and the count of alternative allele X, a binomial model was used to compute the *P*-value for the event that more than X alternative alleles were observed when the null hypothesis is true. When a position is covered by both forward and reverse reads, we used the Stouffer's Z-score method (weighted by the read depths) to combine *P*-values of individual tests into a single *P*-value.

#### Step 3: determination of significance threshold

We ranked the detected candidate mutated positions according to *P*-values. We did not use the nominal *P*-values to call the significant mutations. Instead, we relied on the calibration sample to derive the thresholds. Since there is an imbalance between the number of true positives (calibration SNPs) and true negatives (invariant bases), we used Matthew's correlation coefficient (MCC) [29] to determine the best threshold that maximizes MCC phi coefficient. This non-parametric procedure used in machine learning is optimal to classify binary information such as distribution between true negative and true positive.

#### Step 4: detection in the tested sample

Similar to the calibration sample, the filters above were used on the tested samples. In addition, we used the following filters to determine whether a position in the tested sample is a significant variant.

##### Filter 5

In order to prevent bias due to coverage discrepancies between the calibration and the tested sample, we rejected all bases of the tested sample with a coverage depth lower than the 5th coverage percentile across all bases of the calibration sample.

##### Filter 6

Using the error rate generated from the calibration sample, we calculated the binomial test *P*-values and ranked the candidate position in the tested sample accordingly. We kept all positions above the *P*-value determined by the MCC threshold obtained in the calibration sample.

##### Filter 7

The local sequence-specific context (high GC, low complexity) can lead to lower base quality or alignment success, that is, systematic noise. For each base sequenced, we used the data from the four patient blood samples to estimate its systematic noise. The significance of the noise was measured with a binomial statistics using the local error rate (fraction of non-reference bases) calculated from 20 adjacent positions (10 upstream, 10 downstream). The *P*-value was Bonferroni-corrected for multiple testing in a single 200-bp amplicon (*P *< 5 × 10^-4^). Positions declared recurrently noisy in three or more control blood samples were removed from the list of candidate variants.

To estimate the stringency of the design and of these successive filters, we examined 71 kb of DNA using 518 amplicons that cover 4,446 mutated positions in the COSMIC database, with 5,271 known cancer mutations. The mutations in each sample were called using the algorithm trained on a calibration sample from the same run. Based on the amplicon design and filtering strategy presented above, we estimate that 70% of the targeted COSMIC positions can be assessed by our current method (Figure S10 in Additional file 1).

### Somatic mutation identification, filtering and annotation

We considered all variants in a cancer sample that passed the statistical assessment described above as potential somatic mutations. We then retained the mutations for which the corresponding position in the germline sample was: 1) highly covered (> 10 reads); 2) not called mutant using the same statistical assessment; 3) did not show strong evidence of an alternative allele (> 20%) that failed statistical assessment based on filter 4. Therefore, while most germline mutations will fall under criterion 2, this last criterion identifies rare cases of obvious germline mutations (20% allele frequency), which failed the statistical assessment in the blood sample.

We identified a total of 32 somatic mutations across the four xenograft samples, of which 63% (43/68) recurred in more than one sample and corresponded to mouse-human mismatches. An investigation revealed that a fraction of the PCR primers amplified both human and mouse DNA and that these mutations corresponded to mismatches in the mouse-human genome multiple alignment (Figure S6 in Additional file 1). To filter out false positive mutations in the xenograft samples coming from mouse DNA contamination, we obtained the multiple-alignment Mouse-Human track from the UCSC Genome Browser. We then identified all mismatch positions between hg19 and mm9 genome sequences located in the UDT-Seq amplicons. We filtered out the somatic mutations in the xenograft samples, with coordinates corresponding to these mismatched positions. After removing these exogenous mutations, there were 1, 11, and 1 high confidence somatic mutations in the breast, colon, and ovarian xenograft samples, respectively. No significant somatic mutations were detected in the sarcoma xenograft sample (Table S7 in Additional file 2). To annotate the finalized list of filtered significant somatic mutations for functional consequences, we used ANNOVAR [30] on hg19.

### SNaPshot single base extension assay

For each SNP a region ± 300 bp surrounding the site was downloaded from the UCSC Genome Browser (hg19). SNPmasker 1.0 [31] (hg19 and dbSNP build 132) was used to mask variant alleles in each sequence to avoid introducing allele bias in the PCR primers. PCR primers (Table S10 in Additional file 2) were designed using Primer3. SNaPshot probes (Table S10 in Additional file 2) were designed to anneal adjacent to the mutation site using OligoAnalyzer [32]. Each probe was evaluated for secondary structure formation and designed to have a melting temperature greater than 50°C for the complementary region between the probe and its corresponding template.

PCR was performed in a volume of 25 μl containing 21 μl of Platinum PCR SuperMix (Invitrogen), 200 nM of each primer, and 25 ng template DNA. Samples were amplified using the following cycling parameters: 94°C for 2 minutes, followed by 35 cycles of 94°C for 30 seconds, 55°C for 30 seconds and 72°C for 30 seconds. PCR products were assessed for quality and yield on the NanoDrop spectrophotometer (Thermo Scientific Waltham MA, USA) and by gel electrophoresis on a 2% agarose gel. The remaining 15 μl of PCR amplicons were treated with 5 units of shrimp alkaline phosphatase (SAP) and 2 units of exonuclease I (ExoI) for 1 hour at 37°C, followed by 15 minutes at 75°C to remove excess dNTPs and primers, respectively.

Single base extension reactions (SNaPshot Multiplex Kit, Applied Biosystems) were performed in a volume of 10 μl containing: 5 μl of SNaPshot Mtiplex Ready Mix, 3 μl of SAP/ExoI treated PCR products, 1 μl of 0.2 μM SNaPshot probe, and 1 μl of deionized water. The reactions were carried out in a thermocyler (25 cycles of 96°C for 10 seconds, 50°C for 5 seconds, 60°C for 30 seconds). After extension, the products were treated with 1 unit of SAP at 37°C, followed by 15 minutes at 75°C. Samples were mixed with the GeneScan-120 LIZ size standard and analyzed on the 3130 Genetic Analyzer (Applied Biosystems). We used GeneMapper 3.5 software to analyze the results.

### Sequencing and analysis using Illumina MiSeq

The four calibration sample microdroplet PCR libraries (CAL-A, CAL-B, CAL-C, CAL-D) were amplified using an additional six cycles of universal PCR in the same conditions described above, with the use of a reverse primer containing DNA index (CAAGCAGAAGACGGCATACGAGATXXXXXXGTGACTGGAGTTCAGACGTGTGCTCTTCCGATCTGAC, where X indicates the Illumina index sequence), as recommended by Illumina.

The amplified libraries were purified, quantified and pooled in equimolar amounts. The pool was loaded at 4 pM on one MiSeq flowcell. The sequencing was performed sequentially from both ends each for 154 cycles of chemistry and 151 cycles of imaging, to prevent imaging from the tri-nucleotide PCR-specificity adapters. An additional six cycles were used to read the index. The resulting reads were deconvoluted based on their index. The results were analyzed through the identical analysis pipeline, trimming the reads to 122 nucleotides for comparable statistical analysis. The files corresponding to the raw reads are publicly available at the NCBI Short Read Archive (SRP009487.1) [28].

## Abbreviations

bp: base pair; GAII: Genome Analyzer II; PPV: positive predictive value; SNP: single-nucleotide polymorphism; UDT-Seq: ultra-deep targeted sequencing; WGA: whole genome amplification.

## Competing interests

SKK, JO and DRL are employees of RainDance Technologies. MSC and SP are employees of Prognosys Biosciences. All other authors have no competing interests.

## Authors' contributions

DRL and MSC designed the experiments. OH designed the experiments, designed and performed the analysis and wrote the manuscript. KAF designed the experiments and wrote the manuscript. SKK, JO, SP, and SR performed the experiments. BC provided the samples. LB and KM designed and performed the analysis. RBS provided samples and wrote the manuscript. All authors have read and approved the manuscript for publication.

## Supplementary Material

Additional file 1 **Figures S1 to S10**. Click here for file

Additional file 2 **Tables S1 to S10**. Click here for file
