# Supplementary material for: Detection of low prevalence somatic mutations in solid tumors with ultra-deep targeted sequencing
Source: Genome Biol. 2011 Dec 20;12(12):R124. doi: 10.1186/gb-2011-12-12-r124 (PMC3334619; doi:10.1186/gb-2011-12-12-r124)
Supplement: Additional file 1 — Figures S1 to S10. [file gb-2011-12-12-r124-S1.PDF]

a

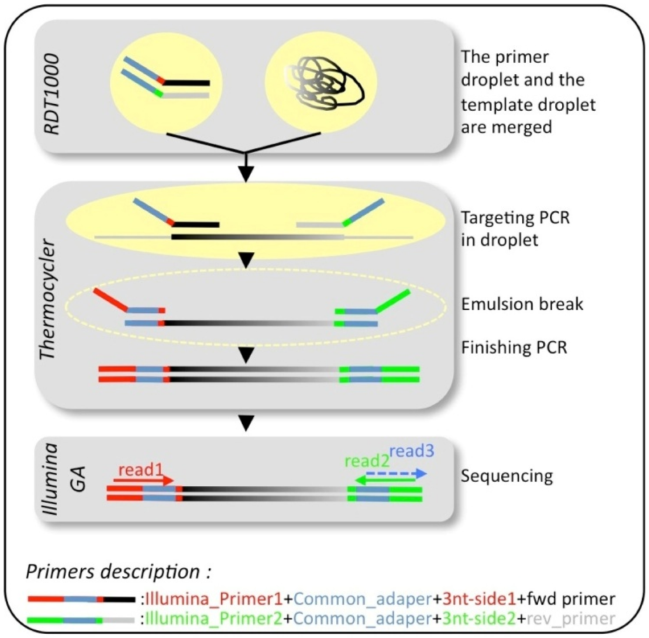

b

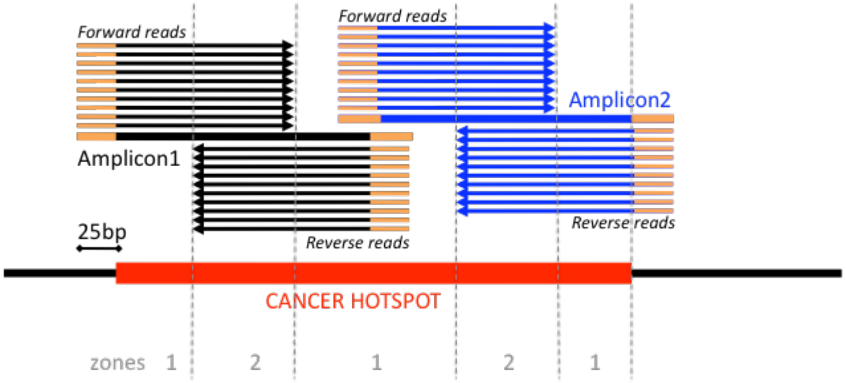

FigureS1: (a) The UDT-Seq laboratory workflow. The DNA samples and the chimeric targeting primers are merged on the RDT1000 instrument. Following the PCR, the emulsion is broken and after purification the resulting sample is re-amplified using universal primers which contain the sequence required for Illumina GAII sequencing. **(b) Principle of Direct Amplicon Sequencing.** Two overlapping amplicons are designed (black and blue) over the cancer hotspot of interest (red). With the help of chimeric primers, the sequencing is primed directly from the 5’ end of the locus specific primer (orange) on both forward and reverse reads (thin arrows). Thus, excluding primer bases, the positions in the cancer hotspots are sequenced either through one strand (forward or reverse, zone 1), two strands (forward and reverse, zones 2) or more strands if the tiles are overlapping by more than half the size of one amplicon.

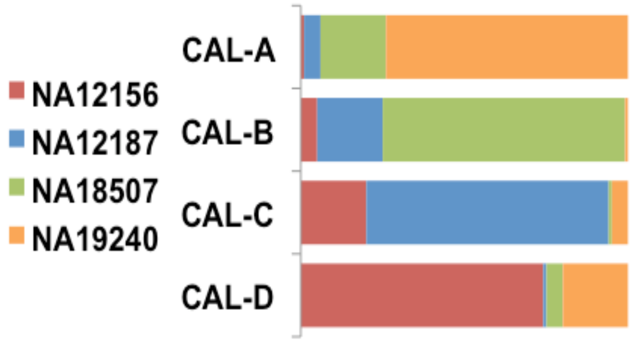

Figure S2: Schematic representation of the relative content of the four Coriell DNA (red, blue, green and orange) in the four calibration samples.

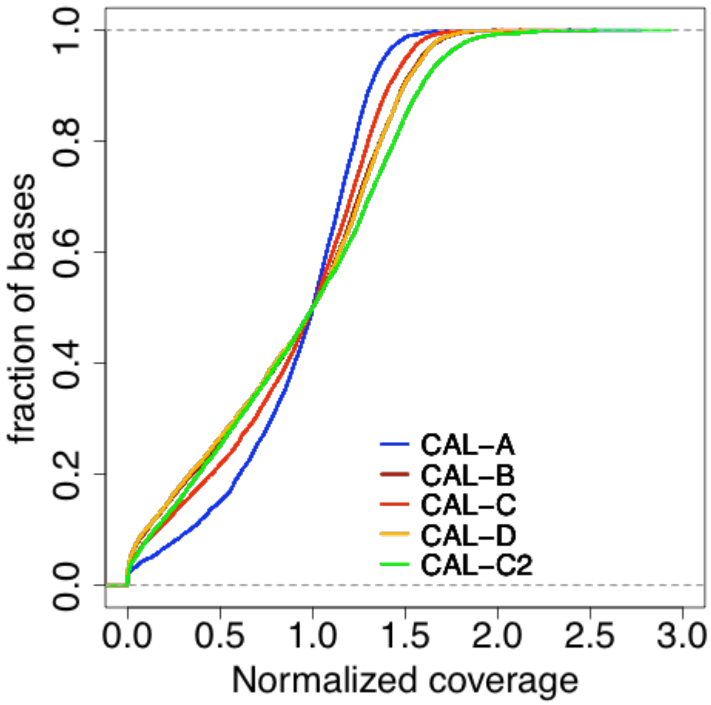

Figure S3: Coverage of the Calibration Sample. Cumulative Distribution of the bases (y axis) at various level of coverage (x axis) for the all sequenced calibration samples.

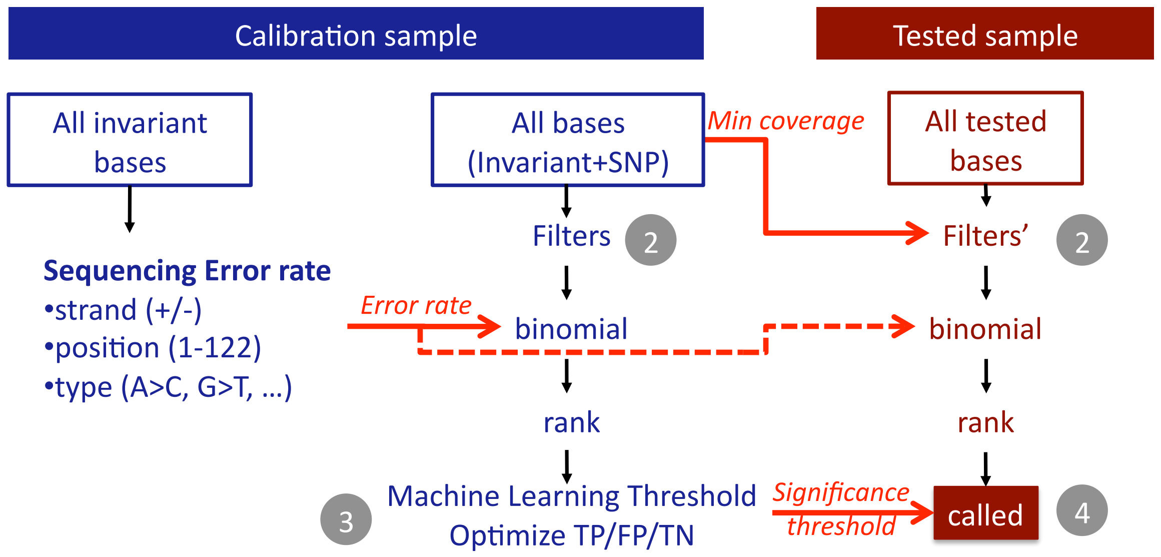

FigureS4: Flow Diagram of the analysis strategy: The four steps described in the main text are indicated in grey. In step 1 the sequencing error rate is estimated using invariant bases in a calibration sample; in step 2, the candidate mutations are filtered and their level of significance is determined in both calibration and tested samples using the error rate; in step 3, the significance threshold is calculated using the known SNPs from the calibration sample; finally, in step 4, the significant mutations are called in the tested sample using this threshold. Values generated in the calibration sample and applied to the tested sample are connected in red. TP: True Positive, FP: False Positive, FN: False Negative

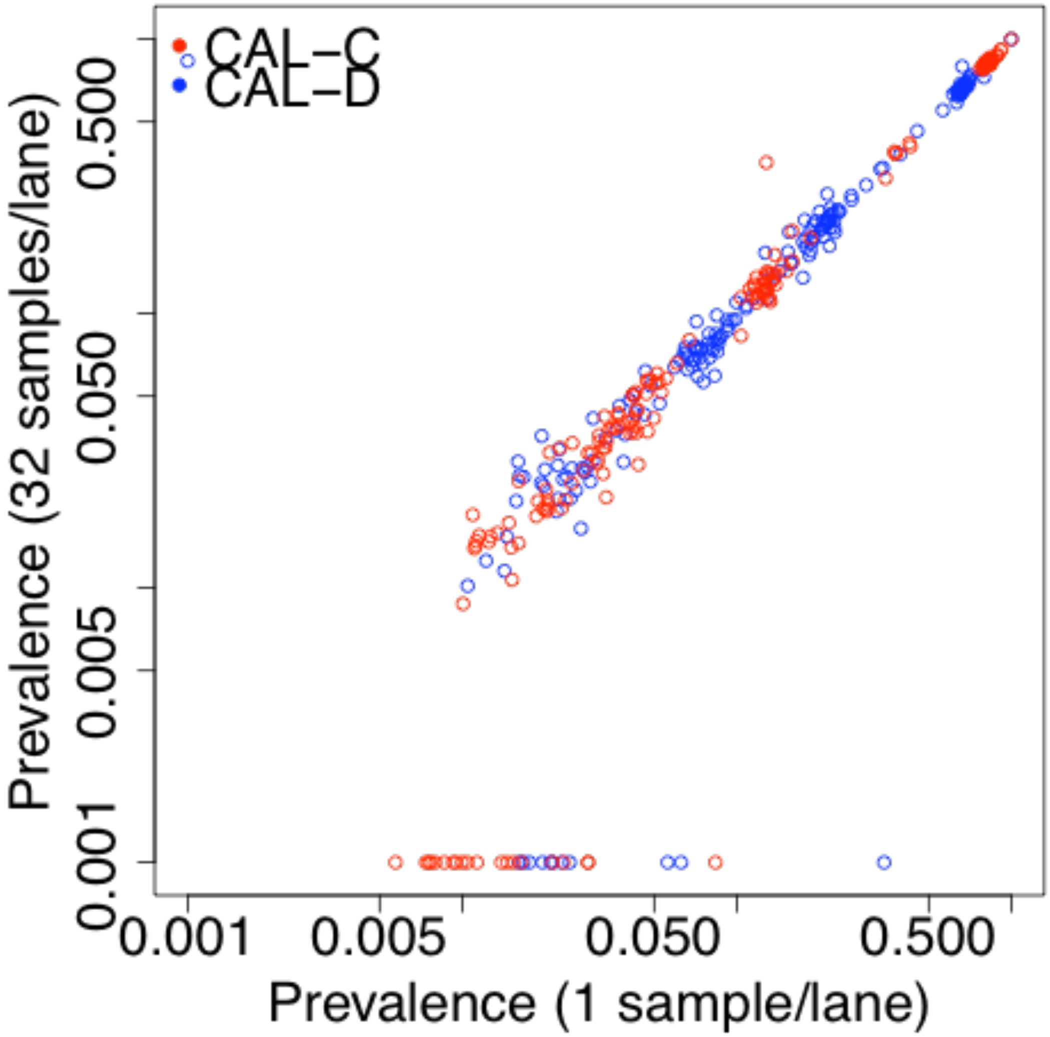

FigureS5: Prevalence observed on multiplexed samples. The prevalence of the calibration SNPs in sample CAL-C and CAL-D simulated at 32 samples per lane (~750x) remains highly correlated with the prevalence at full coverage (~24,000x). Low prevalence SNP are more likely to be missed at lower coverage (arbitrary prevalence of 0.001 for representation purposes).

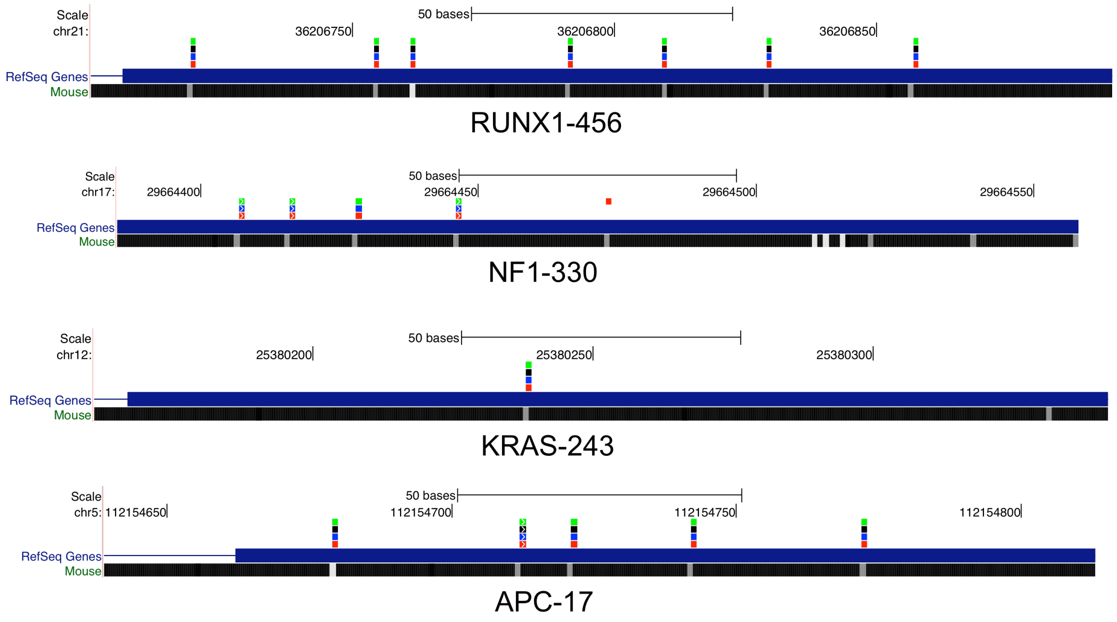

FigureS6: Recurring somatic mutations in the breast (red), colon (blue), ovarian (black) and sarcoma (green) xenograft samples are aligned with the mismatches form mouse/human multiple alignment at four selected amplicons RUNX1-456, NF1-330, KRAS-243, APC-17. A one nucleotide coordinate shift is a representation artifact of the mismatch in the UCSC Genome Browser multiple alignment track.

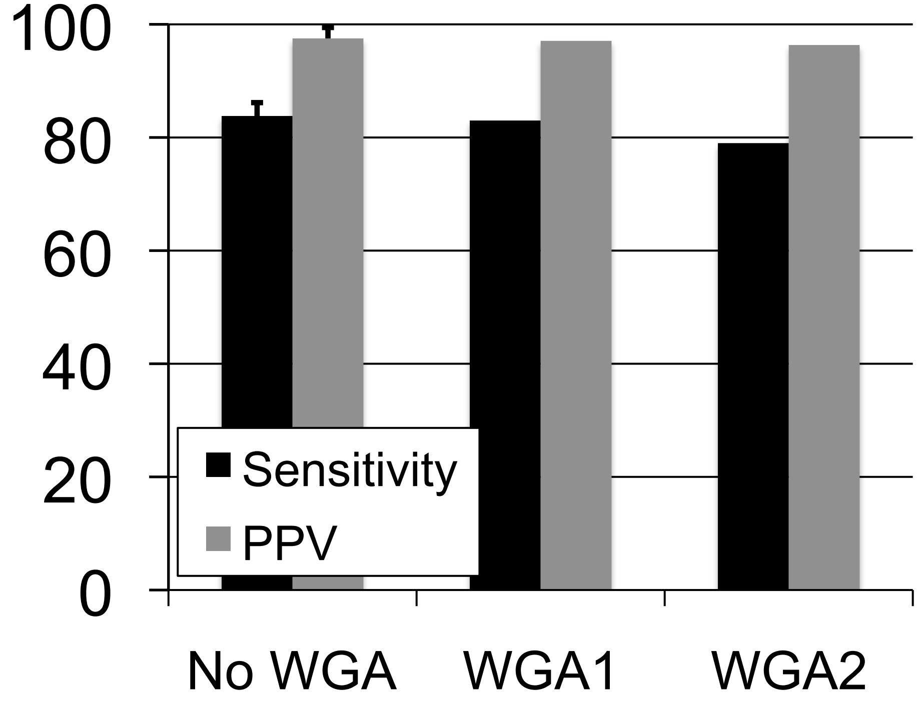

Figure S7: Performance of UDT-Seq after Whole Genome Amplification. The Sensitivity and PPV were calculated on calibration sample CAL-B without amplification (error bars correspond to the standard deviation after training with sample CAL-C and CAL-D) or with whole genome amplification, in duplicate.

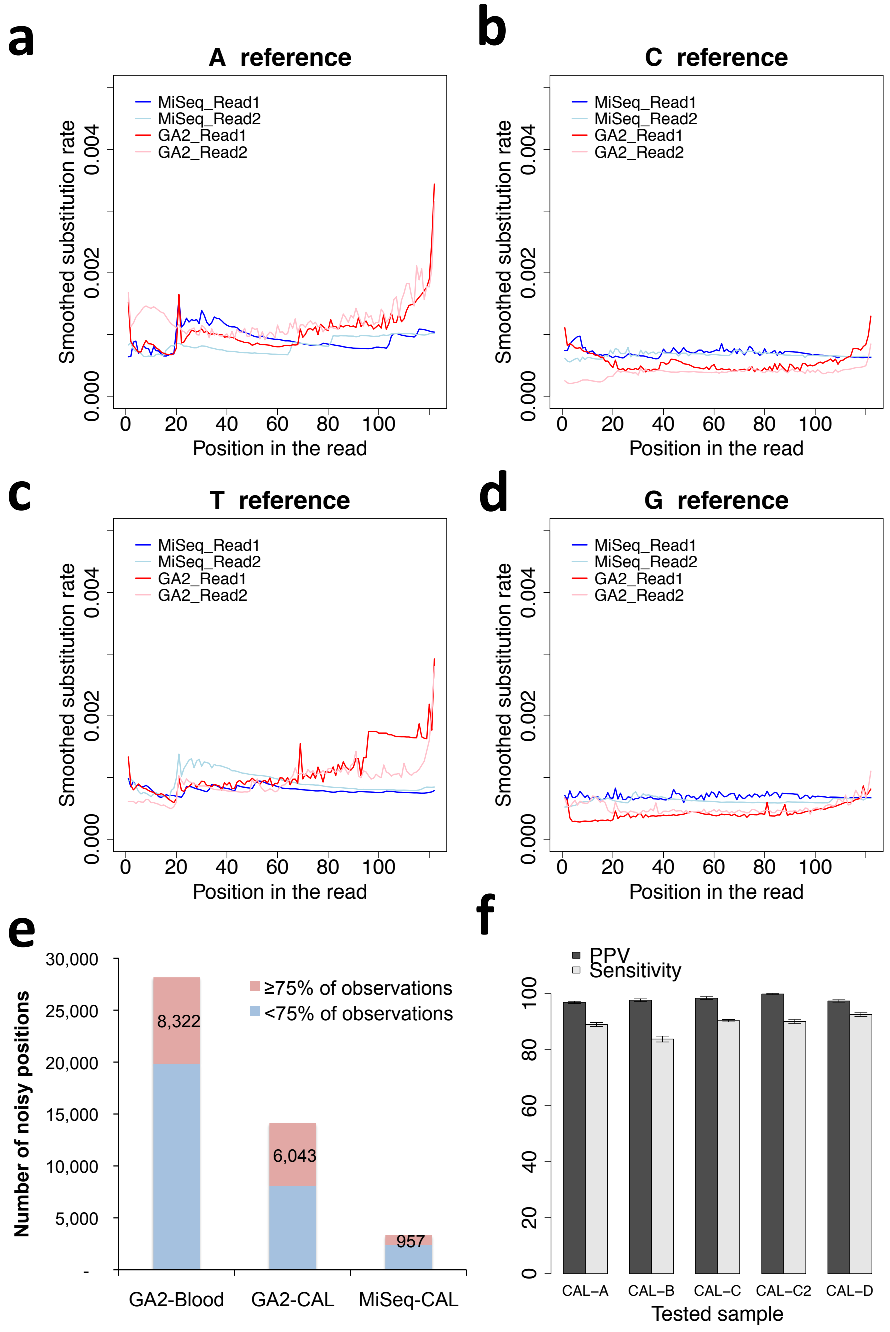

Figure S8: Implementation of UDT-Seq on the MiSeq sequencer. (a) The average smoothed substitution rate is calculated at known invariant “A” reference positions in CAL-A sample as a function of the position in the read (x axis), the instrument used (red: GA2 April 2010, blue: MiSeq Oct 2011) and the sequencing read (dark colors: read1, light color: read2). **(b)** Same as (a) at “C” reference positions. **(c)** Same as (a) at “T” reference positions. **(d)** Same as (a) at “G” reference positions. The first 25-30nt corresponds to the microdroplet PCR primers. **(e)** Number of noisy bases (defined in Filter 7) calculated from the 4 blood samples (only GA2 as described in the study) or from the 4 calibration samples (GA2-CAL and MiSeq-CAL). The positions observed to be noisy more than 75% of the time are indicated in pink and labeled accordingly. **(f)** Summary of the performance metrics obtained from the 4 calibration samples sequenced on the MiSeq. The error bars represent the standard deviation across the various training-testing sample combinations (see **Figure 1a**).

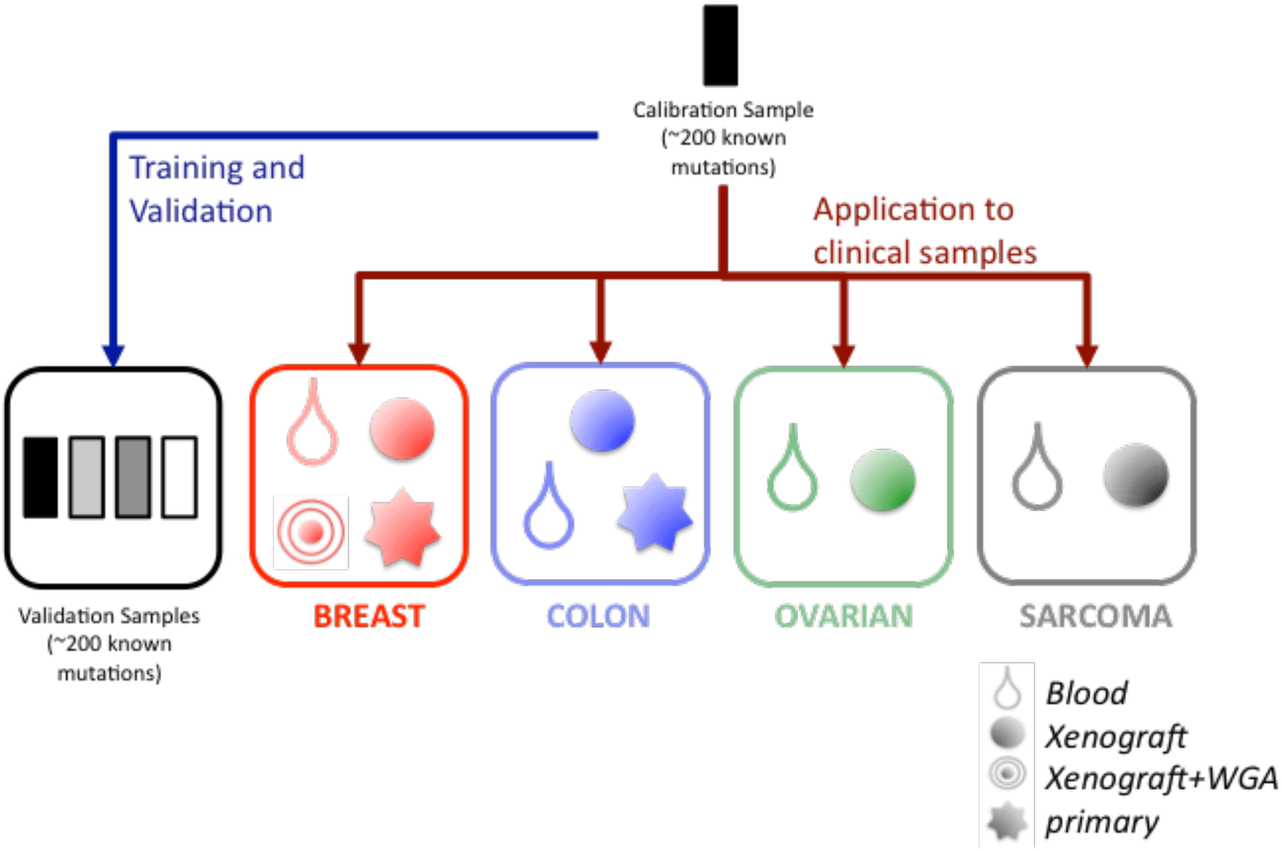

FigureS9: General Overview of the experimental design followed

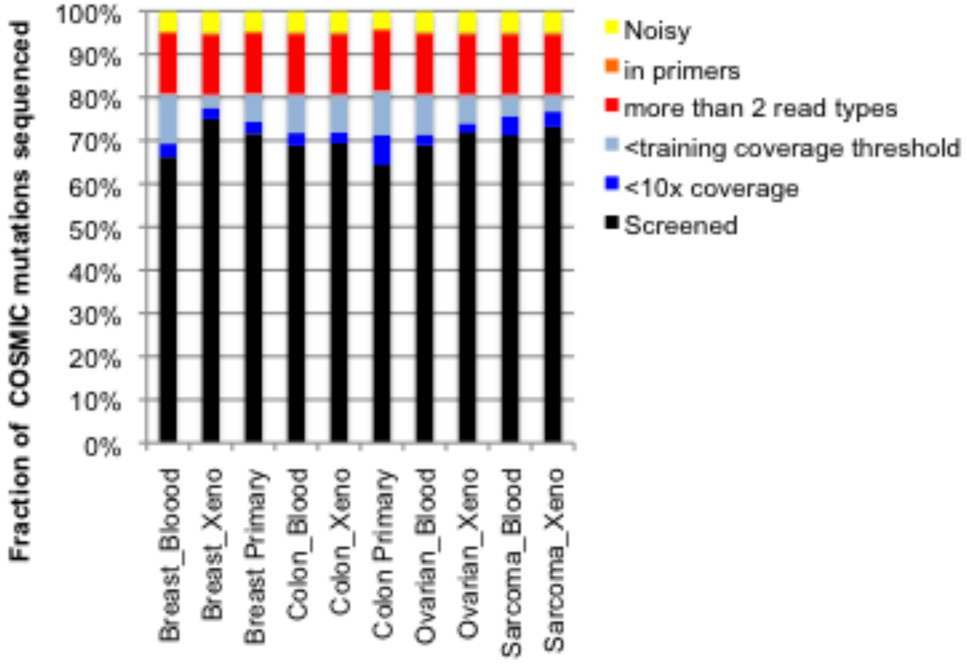

Figure S10: Distribution of the targeted mutations in COSMIC (v44) effectively screened (black) or missed due to design constraints – more than the two read types (forward and reverse) (red) or located in primers (orange) – or due to experimental constraints – noisy position (yellow), low nominal coverage (blue) or coverage lower than the 5th coverage percentile of the calibration sample (light blue).
