# Supplementary material for: Detection of low prevalence somatic mutations in solid tumors with ultra-deep targeted sequencing
Source: Genome Biol. 2011 Dec 20;12(12):R124. doi: 10.1186/gb-2011-12-12-r124 (PMC3334619; doi:10.1186/gb-2011-12-12-r124)
Supplement: Additional file 2 — Tables S1 to S10. [file gb-2011-12-12-r124-S2.PDF]

TableS1: List of the 42 cancer genes screened by UDT-Seq

Gene	No. of COSMIC Mutations[1]	No. of Amplicons
ABL1	21	9
APC	716	44
BRAF	96	6
BRCA1	18	15
BRCA2	33	23
CDH1	124	22
CDKN2A	341	6
CSF1R	12	7
CTNNB1	140	7
EGFR	240	17
ERBB2	35	9
FBXW7	71	16
FGFR1	6	6
FGFR2	7	4
FGFR3	37	10
FLT3	91	11
HRAS	20	3
IKBKB	2	2
JAK2	52	9
JAK3	1	1
KIT	268	14
KRAS	51	3
MAP2K4	14	7
MET	25	11
MLH1	32	17
MSH2	31	13
NF1	104	47
NF2	343	16
NOTCH1	199	15
NRAS	28	3
PDGFRA	48	11
PIK3CA	160	18
PTEN	587	13
RB1	143	27
RET	43	9
RUNX1	88	9
SMAD4	127	16
SMO	17	11
SRC	1	1
STK11	91	12
TP53	230	12
VHL	578	6
**total**	**5271**	**518**

1: Valid mutations only : substitutions or small indels
(<140 bp long) with reported genomic location

Table S2: List of amplicons and primers used in UDT-Seq assay

Chr	Start	End	Amplicon-ID	Forward primers	Reverse primers
chr9	133,729,397	133,729,585	ABL1-1	TCTCCCAATTTTCTCTTCCT	AATCATACAGTGCAACGAAA
chr9	133,730,287	133,730,469	ABL1-2	CAGTCAACAGTCTGGAGAA	CAGTGTTGATCCTGTAATGG
chr9	133,738,195	133,738,394	ABL1-3	GTTCATCATCATTCAACGGT	CTGTATTTCTTCCACACGC
chr9	133,747,361	133,747,549	ABL1-4	CTGTCATGGAACCTGTCT	CTTTCAAGAACTCTTCCACC
chr9	133,748,231	133,748,430	ABL1-5	GTTGTCTTGTTGGCAGG	CCCTACCTGTGGATGAAG
chr9	133,748,233	133,748,432	ABL1-6	TGTCTTGTTGGCAGGG	GCCCCTACCTGTGGA
chr9	133,750,297	133,750,492	ABL1-7	GGTGAAGGTAGCTGATTTTG	AAAAACCCTCCTGTAGTCAG
chr9	133,753,768	133,753,943	ABL1-8	CTTGAACAGCCTTTCTCTTT	AGTTCATAGACCTTCTCTGG
chr9	133,755,350	133,755,549	ABL1-9	AGGATTGGAATGTTGCTTTC	TTTACCGTCTGAGATACTGG
chr5	112,102,020	112,102,212	APC-10	CAGGAAGTACTTAAACAACTACA	CTGATACCAACACCCAAATC
chr5	112,103,030	112,103,207	APC-11	GGTTTGTAAATGGAAGCAGA	TGCAAAATGCTCTAAGTGTT
chr5	112,111,264	112,111,458	APC-12	GCTCTTCTGCAGTCTTTATT	AAGTTGTACTGCCAAGTTAC
chr5	112,116,474	112,116,648	APC-13	TTTGTGGTTTTAGTTTTCCTTACA	TTCCTAATAGCTCTTCGCTGTT
chr5	112,128,053	112,128,233	APC-14	CTGAGCTTTTAAGTGGTAGC	TACTAACCTCTGCTTCTGTT
chr5	112,136,873	112,137,066	APC-15	AATTGATGCATTCAGAGCTT	AAGTTGCCATGTTGATTTCT
chr5	112,151,105	112,151,301	APC-16	ACACTTCATTTGGAGTACCT	TCTTCTGTTACCTTGGTTCC
chr5	112,154,639	112,154,813	APC-17	TTTCTAAACTCATTTGGCCC	CATGTAAAAGCTGGATGAGG
chr5	112,154,823	112,155,017	APC-18	AAGACTCTGTATTGTTGGGA	CTGGTTCATGAGCTTCCT
chr5	112,157,542	112,157,733	APC-19	TTGCTCTTCAAATAACAAAGC	TCCACCAGTAATTGTCTATGT
chr5	112,162,742	112,162,921	APC-20	ACTTGGTACCAGTTTGTTTT	TCAAGTTTGTCAAAGCCATT
chr5	112,163,566	112,163,756	APC-21	AATAAAGCTTGGCTTCAAGT	CTGCCTCAAAGAAAAAGAGA
chr5	112,164,477	112,164,649	APC-22	ACTGCTAGCATTAAAAACAAAA	ATTCCATCAATGCTTTCACA
chr5	112,170,639	112,170,822	APC-23	TCTTACTAGGAATCAACCCTC	GTAATATCCCACCTCCACTT
chr5	112,170,673	112,170,867	APC-24	GTGCCTTATGGAATTTGTCA	TATACCTGTGGTCCTCATTT
chr5	112,173,245	112,173,436	APC-25	TTCAGGCAAATCCTAAGAGA	GTGCTTTGAATGAATGAGGT
chr5	112,173,375	112,173,574	APC-26	AGGAAGCATTATGGGACAT	TTCTAGGGCTTTTTGTTTCC
chr5	112,173,514	112,173,709	APC-27	TATTATGTCTCCTGGCTCAA	ATGTCGATTGGTGTCAAAAA
chr5	112,173,642	112,173,838	APC-28	CTCATCGTAGTAAGCAGAGA	ATCTTTTTCAGAACGAGAACT
chr5	112,173,771	112,173,967	APC-29	ATACTACAGTGTTACCCAGC	TTCCATGACTTTGGCAATC
chr5	112,173,914	112,174,112	APC-30	AAGCGAGGTTTGCAGAT	CCGACTTAGTGAAATTGTAAGT
chr5	112,174,036	112,174,234	APC-31	GACAGATGAGAGAAATGCAC	GGGTTTCATTTGACCTCTTT
chr5	112,174,169	112,174,362	APC-32	TCAAATGATAGTTTAAATAGTGTCAG	GGTGTATCTAGTTCTCCATCA
chr5	112,174,297	112,174,492	APC-33	CGACCTAGCCCATAAAATAC	TTGTCTTTGCTCACTTTGTT
chr5	112,174,429	112,174,628	APC-34	TGAAAGATGGGCAAGACC	TTGTTTCTGAACCATTGGC
chr5	112,174,574	112,174,771	APC-35	CAGCAGGAATGTGTTTCTC	TGTTGGTCTCTCTTCTTCTT
chr5	112,174,708	112,174,902	APC-36	GCCTACCAATTATAGTGAACG	TGTCCAGATGAACTCTTTGA
chr5	112,174,841	112,175,025	APC-37	GCCACAGATATTCCTTCATC	TTTTGAGGCTGACCACTT
chr5	112,174,974	112,175,171	APC-38	AGAATCAGCTCCATCCAAG	GTGTCGTCTGATTACATCCT
chr5	112,175,098	112,175,295	APC-39	ATGTTTTTCAAGATGTAGTTCATT	CTGGATTTGGTTCTAGGGT
chr5	112,175,220	112,175,419	APC-40	AGATTGGAACTAGGTCAGC	ATAGTGTTCAGGTGGACTT
chr5	112,175,365	112,175,549	APC-41	GAAATCTCCCTCCAAAAGTG	GGCTTATAATGCCACTTACC
chr5	112,175,502	112,175,689	APC-42	CCGTTCAGAGTGAACCAT	TCCACTCTCTCTCTTTTCAG
chr5	112,175,620	112,175,813	APC-43	TCAAACAGCTCAAACCAAG	CACTCAGGCTGGATGAA
chr5	112,175,750	112,175,949	APC-44	ACTTTATTACATTTTGCCACG	GCCTCTTTCTCTTGGTTTTC
chr5	112,175,885	112,176,080	APC-45	GACAATGGGAATGAAACAGA	TCTGGGCTGGCTTTTT
chr5	112,175,998	112,176,196	APC-46	TGATGATATTGAAATACTAGAAGAATG	CCCCGGTGTAAAACTAACAT
chr5	112,176,279	112,176,459	APC-47	AATCCCCTCCAAATGAGTTA	ATATCACCTTCCTCTGCTTT
chr5	112,176,509	112,176,708	APC-48	CCTTTCCGTGTGAAAAAGAT	GAGAAAACTCTCTCAGCATT
chr5	112,177,074	112,177,265	APC-49	AGAAACAATCCACTTTTCCC	GCTCAGTCTCTTTGATAGGT
chr5	112,177,767	112,177,966	APC-50	TTACAAGTAATAAAGGCCCAC	AGAGATTGAAGGCATGTTTG
chr5	112,177,803	112,178,002	APC-51	GGGAGAAAAGTACATTGGAA	TCGAACTCCTGGAATATGAA
chr5	112,178,596	112,178,768	APC-52	TGTATTAGTACGCCAGTCAA	GAGACATATCAGGAAGGGAA
chr5	112,178,870	112,179,042	APC-53	GATATTGCACGGTCTCATTC	GCTTTTTCACTGGATTCTGA
chr7	140,439,545	140,439,717	BRAF-54	AAAGTGCTCAGAAATCTGTC	GTCTCCAGATCTCAGTAAGG
chr7	140,453,048	140,453,246	BRAF-55	CAGTGGAAAAATAGCCTCAA	TGCTCTGATAGGAAAATGAGA
chr7	140,477,788	140,477,984	BRAF-56	CACCTGAGTACTCCTACTTC	TAATGGTATGGAGTTAGGGC
chr7	140,481,365	140,481,537	BRAF-57	TACATACTTACCATGCCACT	TTTTCTGTTTGGCTTGACTT
chr7	140,500,110	140,500,303	BRAF-58	AAAGAAAGCGGTTCAAGTAG	TTTGTTTTGTTTTGCCTCAC
chr7	140,508,668	140,508,838	BRAF-59	TTTCACGTCACATACAAACC	TTTGCTCCCTTTACCTCTTA
chr17	41,199,566	41,199,754	BRCA1-60	CAAGAACTGTGCTACTCAAG	CACTTTGAATGCTCTTTCCT
chr17	41,201,093	41,201,267	BRCA1-61	GAGAATATTGTGTCCTCCCT	CCTGGGTTAAGTATGCAGAT
chr17	41,215,268	41,215,460	BRCA1-62	CATTGATGGAAGGAAGCAAA	CCTCTCTATCTCCGTGAAAA
chr17	41,243,606	41,243,793	BRCA1-63	ATCCTGGGTGTTTGTATTTG	TGTCTAAGAACACAGAGGAG
chr17	41,243,980	41,244,152	BRCA1-64	GACTCCTGCTAAGCTCTC	CTTAGAACAGCCTATGGGAA
chr17	41,244,371	41,244,554	BRCA1-65	ATTAATACTGGAGCCCACTT	GCTAGAGGAAAACTTTGAGG
chr17	41,244,736	41,244,929	BRCA1-66	GCTTATCTTTCTGACCAACC	AAATCCAGGAAATGCAGAAG
chr17	41,244,864	41,245,060	BRCA1-67	TGTTTCTTTAAGGACCCAGA	AAGTATCCATTGGGACATGA
chr17	41,245,072	41,245,271	BRCA1-68	TGTCATTTCTATTATCTTTGGAACA	AACTGAAAGATCTGTAGAGAGTAGCA
chr17	41,245,119	41,245,316	BRCA1-69	TTTTCAAATGCTGCACACTG	TGAAGACCCCAAAGATCTCA
chr17	41,245,539	41,245,735	BRCA1-70	TCCATGAGTTGTAGGTTTCT	GCACCTAAAAAGAATAGGCT
chr17	41,246,291	41,246,472	BRCA1-71	TACCTCATTTAGAACGTCCA	CATGCTCAGAGAATCCTAGA
chr17	41,256,161	41,256,359	BRCA1-72	CTCTGTAGAAGTCTTTTGGC	GGGTTTCTCTTGGTTTCTTT
chr17	41,267,717	41,267,905	BRCA1-73	GAGCCACATAACACATTCAA	ACTCAGTCATAACAGCTCAA
chr17	41,275,935	41,276,113	BRCA1-74	ACATGTCTTTTCTTCCCTAGT	ATGGATTTATCTGCTCTTCG
chr13	32,890,554	32,890,748	BRCA2-75	TGCAGACTTATTTACCAAGC	GTGACGTACTGGGTTTTTAG
chr13	32,893,051	32,893,250	BRCA2-76	CGCAAGAGAATGGATTAATGA	AGTTCTTCAAACCAATTAAGACT
chr13	32,900,528	32,900,705	BRCA2-77	TACAGGCAATTCAGTAAACG	TTGACCAAGACATATCAGGA
chr13	32,906,507	32,906,706	BRCA2-78	ACAGTTGTAGATACCTCTGAA	TCAGTATCATTTGGTTCCAC
chr13	32,906,677	32,906,858	BRCA2-79	TGTATCTGAAGTGGAACCAA	GCAATAGGGGTATTTTCTCC
chr13	32,907,330	32,907,529	BRCA2-80	TAGCTTTGAAGAATGCAGG	GGTACCTGAATCAGCATTTG
chr13	32,911,104	32,911,276	BRCA2-81	CAAAAATAACTGTCAATCCAGAC	AACCATGGTAGAGTTCTTGA
chr13	32,911,493	32,911,690	BRCA2-82	TCAAATCACAGTTTTGGAGG	ATTAATTGACTGAGGCTTGC
chr13	32,911,988	32,912,165	BRCA2-83	GTCATAATGAATGCCCCATC	TGCCATGAGCAGAATAAAAG
chr13	32,912,276	32,912,475	BRCA2-84	TCAAGTAAATGTCATGATTCTGT	CTGGCAGCAGTATATTTGTT
chr13	32,912,464	32,912,633	BRCA2-85	TACTGCTGCCAGTAGAAAT	TAATCTGAGTGTTTCCCTCC
chr13	32,912,614	32,912,810	BRCA2-86	GGAGGGAAACACTCAGATTA	TGGCGACACTAATATTTTTCC
chr13	32,912,993	32,913,174	BRCA2-87	AATCAACTAGTGACCTTCCA	TGGCTAAAACTGGTGATTTC
chr13	32,913,154	32,913,323	BRCA2-88	TGAAATCACCAGTTTTAGCC	CCACAGTCTCAATAGAAACAA
chr13	32,913,807	32,914,006	BRCA2-89	TTGAGCCAGTATTGAAGAATGTT	CTCAAAATTATTACTATTAGATATGGACA
chr13	32,913,875	32,914,103	BRCA2-90	AAAGATGCAAATGCATACCC	TGAAACTGTCTGTAAATATGTCTTTC
chr13	32,914,390	32,914,564	BRCA2-91	TAAGTCAGTCTCATCTGCAA	CTGGTCTGAATGTTCGTTAC
chr13	32,929,117	32,929,310	BRCA2-92	CAGTTTCAGGACATCCATTT	ATGTCCATCAATGTTTTGCT
chr13	32,944,444	32,944,626	BRCA2-93	AGGCAGTTCTAGAAGAATGA	ATGATAAGGGCAGAGGAAAA
chr13	32,945,023	32,945,198	BRCA2-94	CCTGGCCTGATACAATTAAC	AGGCTTCTAGTCTCTTTTGT
chr13	32,953,330	32,953,527	BRCA2-95	TAACCACACCCTTAAGATGA	TGAGCTTGTTTCTTATCATTCA

Chr	Start	End	Amplicon-ID	Forward primers	Reverse primers
chr13	32,968,833	32,969,021	BRCA2-96	CCCTTTCGTCTATTTGTCAG	TTTGGACTAGCAGAAAACAC
chr13	32,972,606	32,972,804	BRCA2-97	CTCCTCAGATGACTCCATTT	CGTCGTTTCAGTCTGAGATA
chr16	68,835,574	68,835,754	CDH1-100	GAATTTTGAAGATTGCACCG	CGTGACTTTGGTGGAAAA
chr16	68,835,630	68,835,819	CDH1-101	GATTCAAAGTGGGCACAG	TTCTCAGAAAAATGCCAACA
chr16	68,842,302	68,842,501	CDH1-102	TGTTCCTCATCTTCTTTCCT	TCCCAGAGAAACAGAGAAC
chr16	68,842,622	68,842,805	CDH1-103	CAAGGTTTTCTACAGCATCA	AAAATCCTGGGTGGATGTTA
chr16	68,844,092	68,844,291	CDH1-104	TCTTTCAGCTCTTCTCTCAC	AACCTAAGAGTCTTTCTGAGT
chr16	68,845,575	68,845,774	CDH1-105	GAACTCTTCCAGGAACCTC	CTGACCCCTGACCTCT
chr16	68,845,586	68,845,785	CDH1-106	GGAACCTCTGTGATGGAG	TCTGGATCCTCCTGACC
chr16	68,846,013	68,846,208	CDH1-107	CTTGGTTGTGTCGATCTCT	AAGACCTTTCTTTGGAAACC
chr16	68,847,216	68,847,397	CDH1-108	TACAAGGGTCAGGTGC	TTTGCTGTTTTCAAAATGCC
chr16	68,847,231	68,847,415	CDH1-109	CCTGAGAACGAGGCTAAC	CCAGGTACCATACAAACCTT
chr16	68,849,395	68,849,584	CDH1-110	ACTTCATTGTTTCTGCTCTC	GACACTTCCACTCTCTTTTC
chr16	68,849,488	68,849,685	CDH1-111	TCTCTCTCACCACCTCC	AGTTGAAAAATCCTCACACTT
chr16	68,853,183	68,853,376	CDH1-112	ATATCGGATTTGGAGAGACA	ACTAGCTAGGAGGTCGAG
chr16	68,855,895	68,856,089	CDH1-113	TTCTCTTAGGTTCTCCAGTTGC	CGTGTGTTAGTTCTGCTGTGA
chr16	68,855,942	68,856,128	CDH1-114	ATCCTGTCTGATGTGAATGA	TTGGGTCGTTGTACTGAAT
chr16	68,857,296	68,857,488	CDH1-115	CTCCAGCCCAAGAATCTAT	GGAATTTGCAATCCTGCTT
chr16	68,857,336	68,857,524	CDH1-116	CTTAGAGGTGGGTGACTAC	AAAGCAAGAATTCCTCCAAG
chr16	68,862,067	68,862,255	CDH1-117	ACCATCCCAGTTCTGATTC	TCCGAATAAAGAGATCACCA
chr16	68,863,549	68,863,743	CDH1-118	CTCCAAAGGACTTTGACTTG	TTAGAGATGAGCCATGCTT
chr16	68,867,164	68,867,339	CDH1-119	AAAAGATGCTTTTGTCCCTT	CAAGTAGTCATAGTCCTGGT
chr16	68,771,227	68,771,396	CDH1-98	TTGCGGAAGTCAGTTCA	GTCCCTCGCAAGTCAG
chr16	68,772,173	68,772,364	CDH1-99	GTTCCATCTACCTTTCCCC	TTTCCAACCCCTCCCTA
chr9	21,968,172	21,968,344	CDKN2A-120	CTTCGGTGACTGATGATCTA	AAGTATTTCAATGCCGGTAG
chr9	21,970,879	21,971,085	CDKN2A-121	TTCTCAGATCATCAGTCCTCAC	GGACACGCTGGTGGTG
chr9	21,970,968	21,971,156	CDKN2A-122	CAGGTACCGTGCGACATC	GCGGAGCCCAACTGC
chr9	21,971,034	21,971,233	CDKN2A-123	ATCGCGCACGTCCAG	TCTGACCATTCTGTTCTCTCTG
chr9	21,974,663	21,974,811	CDKN2A-124	GACCCTCTACCCACCT	GGGAGCAGCATGGAG
chr9	21,974,661	21,974,865	CDKN2A-125	CAGACCCTCTACCCACCTG	GGAGAGGGGGAGAGCAG
chr5	149,433,625	149,433,794	CSF1R-126	CAACTCCTCAGCAGAACT	CCCTCAGGACTATACCAATC
chr5	149,436,842	149,437,037	CSF1R-127	CTACTCACATTCTTGGAAGC	TGCTTGATAACAAGGGAAAA
chr5	149,439,236	149,439,414	CSF1R-128	TTTTTCTTGTCCTTTGCCAG	GTCATCACGGAGTACTGTT
chr5	149,441,169	149,441,368	CSF1R-129	CACAGGTCCCTTAAGTCC	GGGCAACAGTTATACTTTCA
chr5	149,449,739	149,449,934	CSF1R-130	ACTTACCTATCAGTGTGGC	AAGTAGGTACTGGGAGATTG
chr5	149,452,930	149,453,110	CSF1R-131	AGGTAGGTCCAGTTAAAACC	TAAACAAGGACATCTTCCCA
chr5	149,459,738	149,459,912	CSF1R-132	AGAAGGAGTAGTTGGTGTG	TCCTCTACTCTAGACCCTG
chr3	41,265,440	41,265,611	CTNNB1-133	GTATCCCAGTGACTTAGGAG	GAGCCCCAATTCAGTAACTA
chr3	41,265,987	41,266,179	CTNNB1-134	ACTAATGCTAATACTGTTTCGT	GTATCCACATCCTCTTCCTC
chr3	41,266,054	41,266,247	CTNNB1-135	CAGAAAAGCGGCTGTTAG	TACCAGCTACTTGTTCTTGA
chr3	41,266,757	41,266,946	CTNNB1-136	AGTTTCAGAATGTCTACCCA	TTTGTATTCTGCATGGTACG
chr3	41,267,020	41,267,195	CTNNB1-137	ATCTTTAAGTCTGGAGGCAT	TGGAGAGTTGTAATGGCATA
chr3	41,267,176	41,267,357	CTNNB1-138	TATGCCATTACAACTCTCCA	CTTACCTTGCTTTCTTGGTT
chr3	41,268,641	41,268,827	CTNNB1-139	TCTCAGACATGTGATCAAGA	CGGCTTATTACTAGAGCAGA
chr7	55,209,965	55,210,146	EGFR-140	TCTTTTTCTTCCAGTTTGCC	AATCAAAGTCACCAACCTTT
chr7	55,210,937	55,211,123	EGFR-141	AGACCTTGAGTTCTTGAGTT	TAAGACTGCTAAGGCATAGG
chr7	55,220,236	55,220,434	EGFR-142	CAGTGACCAAAATCATCTGT	AAGTCTTCTGTCCTGGTG
chr7	55,221,657	55,221,842	EGFR-143	TGAGTGTACTTACCTCACTT	GACACTTCTTCACGCAG
chr7	55,223,528	55,223,725	EGFR-144	TATGTGGTGACAGATCACG	GAAGATGTGTTCCTTTGGAG
chr7	55,232,972	55,233,164	EGFR-145	GGGACCAGACAACTGTATC	GGAAATGTTCTGTTCTCCTT
chr7	55,240,624	55,240,821	EGFR-146	CATGGAATCTGTCAGCAAC	TCACCTCCCTCTCCTG
chr7	55,241,586	55,241,785	EGFR-147	CTTGTCTCTGTGTTCTTGTC	CATGAGAGGCCCTGC
chr7	55,242,396	55,242,581	EGFR-148	CCTTCTCTCTCTGTCATAGG	CAGACATGAGAAAAGGTGG
chr7	55,248,970	55,249,155	EGFR-149	CTCTCCCTCCCTCCAG	CACCAGTTGAGCAGGTA
chr7	55,248,985	55,249,184	EGFR-150	GGAAGCCTACGTGATGG	CTTCCCTGATTACCTTTGC
chr7	55,259,393	55,259,584	EGFR-151	AGGGTCTTCTCTGTTTCAG	TAAAGCCACCTCCTTACTTT
chr7	55,259,405	55,259,586	EGFR-152	GTTTCAGGGCATGAACTAC	CCTAAAGCCACCTCCTTAC
chr7	55,260,370	55,260,549	EGFR-153	CACTCGTAATTAGGTCCAGA	AGGATTATGACTCACCGTAG
chr7	55,266,348	55,266,534	EGFR-154	CAAGGGATTGTGATTGTTCA	ATCGATGGTACATATGGGTG
chr7	55,268,862	55,269,060	EGFR-155	CTCAAAATCTCTGCACCAG	GAGATTTCATACCAGAGAGC
chr7	55,269,324	55,269,502	EGFR-156	ACCTTCACAATATACCCTCC	TCTGGCTTATAAGGTGTTCA
chr17	37,863,162	37,863,360	ERBB2-157	TGGAGTGAGTTTGGATGG	TAGGTGAGTTCCAGGTTTC
chr17	37,866,266	37,866,459	ERBB2-158	CTAGAAGGTGATGCTGATGA	CATACCAGGCAGTCAGAG
chr17	37,868,166	37,868,347	ERBB2-159	TCCTGATCTCCTTAGACAAC	CAGAGGGACAGGAACTG
chr17	37,879,844	37,880,021	ERBB2-160	GATCCTGAAAGAGACGGAG	CCAAACACTGCCTCCA
chr17	37,880,180	37,880,350	ERBB2-161	GATGGGGAGAATGTGAAAAT	AAACCCCAATGAAGAGAGA
chr17	37,880,971	37,881,159	ERBB2-162	GTCCCCAGGAAGCATAC	CAATCTGCATACACCAGTTC
chr17	37,881,305	37,881,474	ERBB2-163	ATGAGCTACCTGGAGGAT	TTGGTCCTTCACCTAACC
chr17	37,881,457	37,881,654	ERBB2-164	GGTTAGGTGAAGGACCAA	ATAACTCCACACATCACTCT
chr17	37,881,898	37,882,089	ERBB2-165	TCTGGCTCAGTACACTAAAG	TAGACATCAATGGTGCAGAT
chr4	153,244,063	153,244,253	FBXW7-166	CAGCAGCTTGGTTTCTTC	ACTTTGTAATTACCAGCTCAG
chr4	153,244,114	153,244,313	FBXW7-167	CTTTGTGTTTGAGGCTCTG	GTTTTTGTCTAGGTCCCAAC
chr4	153,245,343	153,245,512	FBXW7-168	TGTTTGTAAACACTGTCCTG	ATACATCAATCCGTGTTTGG
chr4	153,247,124	153,247,331	FBXW7-169	GGAGAGCATTTAAGGGAGAGA	GGCCAGTGTTTACATGTTTTG
chr4	153,247,200	153,247,386	FBXW7-170	TAGACAGGTTTCAGTCTCTG	CAGAGTTGTTAGCGGTTC
chr4	153,249,338	153,249,537	FBXW7-171	CCATGACAAGATTTTCCCTT	TGAGAACATTAGTGGGACAT
chr4	153,249,342	153,249,541	FBXW7-172	GACAAGATTTTCCCTTACCTT	TGTCTGAGAACATTAGTGGG
chr4	153,250,833	153,251,029	FBXW7-173	GACTGCTGACCAAACTTTTA	GTTCCTGTTTATGCCTTCAT
chr4	153,251,780	153,251,958	FBXW7-174	CTACAGAAGAGGAGTGTCAT	CCATGGAAAAGTGCATACAT
chr4	153,253,690	153,253,877	FBXW7-175	CAGAATCACTCTGCTTTTCA	TCTTAGTTGGCACTCTATGT
chr4	153,258,947	153,259,143	FBXW7-176	TCTTTACCTCTTTAGGGAGC	CCTGTAATTTGGGACATCTG
chr4	153,268,037	153,268,212	FBXW7-177	AGCAATTAAGTGAGGCATTT	TTGTACCATGTTCAGCAAC
chr4	153,271,076	153,271,275	FBXW7-178	GTGTTAAACAGTCAACCGTA	TGAAAAGAAAGTTGGACCAT
chr4	153,332,469	153,332,661	FBXW7-179	TATAGAATGGGGAGGAGAGT	GAGGAAGATGAAGAACATGC
chr4	153,332,504	153,332,684	FBXW7-180	TCCACAATACTACTGGAGTT	CCTCAGGAAACCAAGAAGA
chr4	153,332,859	153,333,028	FBXW7-181	TCTGTTCTTCATCTACCTGG	GCAGAATGTGAAAACCTTTG
chr8	38,272,068	38,272,264	FGFR1-182	CAAACTCACACATCACTCTG	GTTTCATCTGAGAAGCAAGG
chr8	38,272,249	38,272,435	FGFR1-183	GCTTCTCAGATGAAACCAC	CTTCCTCCTTCCTCAGTG
chr8	38,273,380	38,273,573	FGFR1-184	TTCCACACCTTCTTGGAG	TTGTATGTCATCGTGGAGTA
chr8	38,274,688	38,274,887	FGFR1-185	CACATTTTAAACCTCTGCCA	ATGATGAAGATGATCGGGAA
chr8	38,282,129	38,282,326	FGFR1-186	CTTACACATGAACTCCACG	AGGTTTACAACCCATCACT
chr8	38,285,833	38,286,017	FGFR1-187	AAGGGCAGTAAGATAGGAAA	ACTAGCCTTGGTGAAATCTA
chr10	123,255,972	123,256,150	FGFR2-188	TCCAAATTGCCTGTTTTCTT	TCCTATGACATTAACCGTGT
chr10	123,279,528	123,279,708	FGFR2-189	CGTATTTACTGCCGTTCTTT	CTCCTTTCTTCCCTCTCTC
chr10	123,298,119	123,298,288	FGFR2-190	GTGGTACGTGTGATTGATG	AGTACTTGGTATTCTGTGCT
chr10	123,310,796	123,310,966	FGFR2-191	AATTCTACCTTGTAGCCTCC	ATACTGGACCAACACAGAAA
chr4	1,801,086	1,801,284	FGFR3-192	CTGTCTGGGTCAAGGATG	TCCTTTCTGTAGCTGGC
chr4	1,803,371	1,803,570	FGFR3-193	GTCATGGAAAGCGTGGTG	GGGAGCGCTCTGTGG

Chr	Start	End	Amplicon-ID	Forward primers	Reverse primers
chr4	1,803,515	1,803,699	FGFR3-194	TGGTGGTGAGGGAGG	CCTCCACGTGCTTGAG
chr4	1,805,348	1,805,541	FGFR3-195	CCGTGTGGACTCTGTG	AGAGTGATGAGAAAACCCAA
chr4	1,805,522	1,805,720	FGFR3-196	TTGGGTTTTCTCATCACTCT	ACTTTGTCCCCACACTG
chr4	1,806,063	1,806,261	FGFR3-197	AGGAGCTGGTGGAGG	CTACTTTCTGTTACCTGTCG
chr4	1,806,429	1,806,622	FGFR3-198	ATCTTCATTCAATGCTGGTG	CCCTGAGGACAGCCT
chr4	1,807,768	1,807,944	FGFR3-199	CCTTCCCCAGTGCATC	CTACTGGCATGACCCC
chr4	1,808,156	1,808,354	FGFR3-200	GTGTGGTTTCTACCCCTC	GAAGAGCTCCTCCACAG
chr4	1,808,841	1,809,022	FGFR3-201	AGGAGTACCTGGACCTG	GGACCCCTCACATTGTT
chr13	28,589,241	28,589,436	FLT3-202	GACAGACTGTACCTTTCTGA	AAGAAATGGATGAATGGCTG
chr13	28,592,555	28,592,738	FLT3-203	CACAACACAAAATAGCCGTA	CATTCTTGACAGTGTGTTCA
chr13	28,599,048	28,599,222	FLT3-204	GGGTGTATCTGAACTTCTCT	AAATGGACAAATGGATGGTG
chr13	28,602,261	28,602,445	FLT3-205	TTGTCATCAAGCTACAGTCT	AATGCTCCTTTCTTTGACAG
chr13	28,607,999	28,608,198	FLT3-206	TTGCTGTCCTTCCACTATAC	ATGTTTCTGCAGCATTTCTT
chr13	28,608,112	28,608,309	FLT3-207	TGATCCTAGTACCTTCCCT	GGCTCCTCAGATAATGAGT
chr13	28,608,197	28,608,394	FLT3-208	ATTTGGCACATTCCATTCTT	AGAACTGCCTATTCCTAACT
chr13	28,610,080	28,610,264	FLT3-209	CTTGTCTGAACACTTCTTCC	CCTTGGCTTCACAAAGTATT
chr13	28,622,492	28,622,691	FLT3-210	GGCTTTAAACCTGACAGAAA	CACTTGAGTTTTCCTGTGTT
chr13	28,623,450	28,623,632	FLT3-211	CCACATACTTCACATTCCAC	ATGATACGGATTCTGTTTGC
chr13	28,626,627	28,626,820	FLT3-212	AGAAAAAGGCCAAAAGGAAA	ATGCTTTAGATACCCTGCTT
chr11	533,437	533,617	HRAS-213	TGGAGAGCTGCCTCA	CTCAGGGAGCAGATCAAA
chr11	533,777	533,963	HRAS-214	GATGTCCTCAAAAGACTTGG	GAGCCCTGTCCTCCT
chr11	534,144	534,333	HRAS-215	CTATCCTGGCTGTGTCC	CCTGAGGAGCGATGAC
chr8	42,174,303	42,174,494	IKBKB-216	TTGAAGGCCAGAATCCAA	TGAAAGTGTGTTGAGTTTCC
chr8	42,179,587	42,179,776	IKBKB-217	GTGACAGTCAGGAAATGGTA	AAACTCTCTTCATGAGGTCC
chr9	5,050,671	5,050,859	JAK2-218	AATTTTTGGTTTTAGTGGCG	TGTGTAAGGATTTGCAAAAGA
chr9	5,069,931	5,070,128	JAK2-219	CAAACCTTCTAGTCTTCAGAAC	ACATGAATGTAAATCAAGAAAACA
chr9	5,072,504	5,072,681	JAK2-220	GGCCAAGGCACTTTTAC	AGAGCACATCTTTAAACAGC
chr9	5,073,702	5,073,872	JAK2-221	TCTTTGAAGCAGCAAGTATG	CTAGCTGTGATCCTGAAACT
chr9	5,077,361	5,077,560	JAK2-222	AATGGTCACATGTAAGTATAAAGA	CCAACTGTTTAGCAACTTCA
chr9	5,078,336	5,078,516	JAK2-223	TGTGCCAAAAATATTCTGCT	TAACAACATGCCCTTTACAC
chr9	5,081,663	5,081,833	JAK2-224	TCAGTTTAGTCCAGAGAATGT	TGTCTCTCTTCAAACTGTGT
chr9	5,089,647	5,089,834	JAK2-225	TCCATCCTAATGTGATGTGT	ACAATGTTGTCATGCTGTAG
chr9	5,126,626	5,126,799	JAK2-226	CCAATTAAAAGATGGCCCTT	CATTTCTTTCATCCAGCCAT
chr19	17,948,833	17,949,032	JAK3-227	GGTAAATCTTGGTGAAGGAC	GGTCCAGAGACTCAGATG
chr4	55,561,725	55,561,920	KIT-228	ATCCATCCAGGAAAATCAGA	TTAAGCCGTGTTTGTTGG
chr4	55,589,665	55,589,841	KIT-229	GTTGTAGGGATTAGAGAGGG	ATACCAATCTATTGTGGGCT
chr4	55,592,018	55,592,227	KIT-230	TTTAGATGCTCTGCTTCTGTACT	AAGAAATATACCTTTGTTGTTACCTTT
chr4	55,592,047	55,592,246	KIT-231	GGATGTGCAGACACTAAACTCA	CCCCTTAAATTGGATTAAAAAGA
chr4	55,593,412	55,593,611	KIT-232	TCCTTTGCTGATTGGTTTC	AACCTTCCACTGTACTTCAT
chr4	55,593,548	55,593,745	KIT-233	AAAAGGTGATCTATTTTTCCCT	AAAAAGGTGACATGGAAAGC
chr4	55,593,924	55,594,097	KIT-234	CAAATGGTCCTTCAATTCCA	TTACGCTTGAGCATCTTTAC
chr4	55,594,151	55,594,324	KIT-235	GCTAAAATGCATGTTTCCAA	TGACAGACAATAAAAGGCAG
chr4	55,595,492	55,595,690	KIT-236	TGCTTTTAGGGCCCAC	GCCTGTCAACAGCTAACTA
chr4	55,597,397	55,597,587	KIT-237	TTATGTAGCAAAGGGGATGA	ACCTATTCTCACAGATCTCC
chr4	55,598,016	55,598,186	KIT-238	AAAATCCTCTCTTCCTCACA	TTAGAGAATCACTCCCACTT
chr4	55,599,211	55,599,386	KIT-239	TTTCTTTTCTCCTCCAACCT	GACTGTCAAGCAGAGAATG
chr4	55,602,639	55,602,829	KIT-240	TGACTCTGTTGTGCTTCTAT	TTCAAGAAGATGCTCTGAGT
chr4	55,603,275	55,603,444	KIT-241	TGCCCATACATTTGAAAACA	GATTGGTGCTCTCTGAAATC
chr12	25,378,543	25,378,720	KRAS-242	CTTACCTGTCTTGTCTTTGC	TTTTCTTTCCCAGAGAACAAAT
chr12	25,380,161	25,380,342	KRAS-243	AACCCACCTATAATGGTGAA	CCTACAGGAAGCAAGTAGTA
chr12	25,398,156	25,398,328	KRAS-244	GTATCAAAGAATGGTCCTGC	CCTGCTGAAAATGACTGAAT
chr17	11,998,803	11,999,002	MAP2K4-245	CGGTTTTTCTCTACCATGAG	GAGTGCACCATAAAACTGAA
chr17	12,011,113	12,011,301	MAP2K4-246	TGTTGGATCTGTATGGAACT	ATGTGCATTCAAAACTCCAG
chr17	12,013,588	12,013,762	MAP2K4-247	AATGTATGCAGAGGACTACA	AATACCAATCCATACCCACC
chr17	12,016,476	12,016,649	MAP2K4-248	TTTTGCTTAAAGTGAAGCCT	CTTGTCTTGGCAATAGAGTC
chr17	12,028,512	12,028,711	MAP2K4-249	ATATACTGGCATTTTGGCTG	AGGTTGAATCAGCATAAACA
chr17	12,032,332	12,032,529	MAP2K4-250	CCTGTGTTTAATTCAAGGCT	TTCACGACTTGTGTTAGTTG
chr17	12,043,044	12,043,234	MAP2K4-251	TGGATCTGAAGGAAAGAACT	ATGTACCTACAATCCCCAC
chr7	116,339,621	116,339,814	MET-252	ATTCTCCCCACAGATAGAAG	AACCATCTTTCGTTTCCTTT
chr7	116,339,691	116,339,876	MET-253	CTTTCATCTGTAAAGGACCG	GGGGTAAGAATCTCTGAACT
chr7	116,340,121	116,340,298	MET-254	AGCTTGCTAGACAAATAGGA	TGGAGACATCTCACATTGTT
chr7	116,380,897	116,381,085	MET-255	TCTTCACAGATCACGAAGAT	TCCTACCTTGTAGATTGCAG
chr7	116,411,900	116,412,098	MET-256	AAGATCTGGGCAGTGAATTA	CAACCCACTGAGGTATATGT
chr7	116,417,361	116,417,535	MET-257	TGAAGCTCATAAAGGGTTTG	AATGCCACTTACTGTTCAAG
chr7	116,418,826	116,419,024	MET-258	TCAGGAATCACTGACATAGG	GCAGTCAACTTACATGAGTC
chr7	116,418,848	116,419,047	MET-259	AAGTTTCCCAATTTCTGACC	TAGTTTGCCAGTTAGTAAGC
chr7	116,422,036	116,422,210	MET-260	CTGCAGAATCCAACTGTAAA	GAAACAGATTCCTCCTTGTC
chr7	116,423,356	116,423,554	MET-261	AGGCTGGATGAAAAATTCAC	AGGAGAAACTCAGAGATAACC
chr7	116,435,680	116,435,855	MET-262	CTCATCTGTCCTGTTTCTTG	GACTACTTACAAGGGGTCTG
chr3	37,038,028	37,038,227	MLH1-263	GTTATCATTGCTTGGCTCAT	ATCCTGCTACTTTGAGGTTT
chr3	37,042,378	37,042,567	MLH1-264	TGGGAATTCAAAGAGATTTGG	CATTTCTTGAATCTTTAGCTTACC
chr3	37,048,410	37,048,579	MLH1-265	ATTTTCTCTTTTCCCCTTGG	TTACTCTCCCATGTACCATT
chr3	37,050,288	37,050,475	MLH1-266	ATCAATCTTCTGTTCAGGTG	ACTGTTCAATGTATGAGCAC
chr3	37,053,522	37,053,701	MLH1-267	TGTTAGGACACTACCCAATG	AAGCAAACTCTTAACACACA
chr3	37,055,896	37,056,078	MLH1-268	AAAGCTTCAGAATCTCTTTTCT	GTTTCCTGTGAGTGGATTTC
chr3	37,058,963	37,059,152	MLH1-269	GTTTTGAACTGGTTGCTTTC	GAGAGCCTGATAGAACATCT
chr3	37,061,751	37,061,944	MLH1-270	CTCCCCCTCCCACTATCTAA	TACATCCTGGAGGAATTGGA
chr3	37,067,217	37,067,392	MLH1-271	TAAGGTCTATGCCCACCAGA	GGAGTTCAAGCATCTCCTCA
chr3	37,067,368	37,067,566	MLH1-272	CAAGATGAGGAGATGCTTG	CCAAAGTTAGAAGGCAGTTT
chr3	37,070,250	37,070,432	MLH1-273	CTGCACTTCCTTTTCTTCAT	TTTACGTACCCTCATGTCC
chr3	37,081,620	37,081,802	MLH1-274	GGGGTTGGTAGGATTCTATT	ACACTCAGCTGATTTACCTA
chr3	37,083,694	37,083,889	MLH1-275	CCAACTGGTTGTATCTCAAG	AGTGGAGAGCTACTATTTTCA
chr3	37,088,989	37,089,188	MLH1-276	CATGTTCTTGCTTCTTCCTA	AATGGCTGTCACACCTC
chr3	37,089,936	37,090,132	MLH1-277	CTGGAGAAATGGGATTTGTT	CCGAAATGCTTAGTATCTGC
chr3	37,090,376	37,090,565	MLH1-278	TTGAATTTCTTTGGACCAGG	AAGATTGTATGAGGTCCTGT
chr3	37,092,107	37,092,289	MLH1-279	ACCTGCCTGATCTATACAAA	AGGAATACTATCAGAAGGCA
chr2	47,630,444	47,630,640	MSH2-280	CCGGGGCGACTTCTATAC	CACTGGAGAGGCTGCTCA
chr2	47,637,228	47,637,405	MSH2-281	CTTAGGCTTCTCCTGGCAAT	TCAGGGAATTCACACAGTCC
chr2	47,639,500	47,639,692	MSH2-282	TGCTTTTCTTATTCCTTTTCTCA	TCCATTTCTGGCAATACAGC
chr2	47,641,415	47,641,606	MSH2-283	TTTCATCACTGTCTGCGGTA	CCATTCAACATTTTTAACCCTTT
chr2	47,643,362	47,643,554	MSH2-284	CTGTTTTTCATGGCGTAGTA	GTTCTTATCCATGAGAGGCT
chr2	47,656,908	47,657,077	MSH2-285	AGATGCAGAATTGAGGCAGA	CATGTTTTTCCAGAGCCTGT
chr2	47,690,150	47,690,323	MSH2-286	TCACTTTGTTCTGTTTGCAG	TTATTCCAACCTCCAATGAC
chr2	47,693,829	47,694,017	MSH2-287	TCCAGTGCACAGTTTGGATA	AACATTCACATCATGTTAGAGCA
chr2	47,698,045	47,698,241	MSH2-288	CACATTGCTTCTAGTACACA	CAGGTGACATTCAGAACATT
chr2	47,702,172	47,702,371	MSH2-289	GAACCAATGCAGACACTCAA	TATACGTCATTAGGAATAAATGCAA
chr2	47,703,495	47,703,676	MSH2-290	TTGTTTTGTAGGCCCCAAT	TGAACGTGGAGACTCCTTTC
chr2	47,705,408	47,705,578	MSH2-291	CAGGTCTGCAACCAAAGATT	TGATTGGCCAAGGCAGTA

Chr	Start	End	Amplicon-ID	Forward primers	Reverse primers
chr2	47,707,818	47,708,016	MSH2-292	CCCCAAATTTCTTATAGGTGT	ACAAACCTCTCTTTCCAGAT
chr17	29,485,952	29,486,121	NF1-293	TTCACTTTTCAGATGTGTGT	ATTTACTTACCCCAGCAAGA
chr17	29,490,207	29,490,401	NF1-294	CCAAAGGACACAATGAGATT	CACTAACCTGGTAGAAATGC
chr17	29,496,832	29,497,023	NF1-295	GGGATTACAGGTGTGAGATA	AAACTTACCCTTCAGGAGTC
chr17	29,508,375	29,508,569	NF1-296	TGCTCTGAGTTGTATTTGTG	GCTAACAACAGCAAATTTTACA
chr17	29,509,523	29,509,707	NF1-297	CAGAATGTGCAGAAAAGCTA	TGGAAATAATTTTGCCCTCC
chr17	29,527,442	29,527,618	NF1-298	GTTATTTCTGGACAGTCTACG	GTTACCTTAAGATCAACCACC
chr17	29,528,080	29,528,277	NF1-299	CATTCTCAAGAGGCAGTCA	AAATACCGATAAAAGTGAACCT
chr17	29,528,423	29,528,596	NF1-300	CTATAGATCTGCCTGGCTC	TGAAAACCAAGAGTGCATTT
chr17	29,533,243	29,533,428	NF1-301	TTTGTTTTTCTCTAGTCCGC	AGAAAGGAGGTGAGATTCAA
chr17	29,545,960	29,546,159	NF1-302	CTCCTTCTAATCTCTCTCGAT	CATGGAATTCATTTTCCCCT
chr17	29,548,750	29,548,940	NF1-303	ACTACAAATGAAAGAGCTCAA	CCCAAAATGTTTCTACAGGA
chr17	29,552,029	29,552,206	NF1-304	CAAACAGGAAGACAACTCAA	GATCCATGGACATTTGACTG
chr17	29,553,458	29,553,665	NF1-305	TGCAGCAGGATGCAG	AAGAGGTTATGCACTGACA
chr17	29,553,524	29,553,720	NF1-306	GTACATGTTTCTGTGGAACC	ACCACTATTCACATTTACCTG
chr17	29,554,194	29,554,371	NF1-307	GCTCTAAGTGCAGTAACTTG	CAAGAGGCTTCCAAATTGAG
chr17	29,554,495	29,554,665	NF1-308	AGCTCTAGACTAAGTTGCTT	GCAGATCAGTTAACAGACAA
chr17	29,556,047	29,556,231	NF1-309	CTGAAAGCCTTCACAAGAC	GGGTGGGCTATAGGTTG
chr17	29,556,339	29,556,509	NF1-310	GGTGTGTAACCATGAGAAAG	AGGTGAAAAATAAGAGAACACT
chr17	29,556,874	29,557,064	NF1-311	ACTCAATTTGTAGAACAAACCA	ACCAGTATCAGTGTGTAAGA
chr17	29,557,301	29,557,494	NF1-312	TGGTCCATGCAATTCAAATA	CTTTCTACCAATAACCGCAT
chr17	29,557,807	29,557,992	NF1-313	GTGTGGCTTCAAAAACATTG	AACTCTCACAGTAAAACCCA
chr17	29,559,675	29,559,869	NF1-314	ATACGGCCTTCACTATGTAA	TGGCATTGAGTAAGTTTGAC
chr17	29,559,948	29,560,127	NF1-315	GGCATGTAAGAGAAGCAAAA	CCAATACTGTTTCTGCAAGT
chr17	29,562,592	29,562,773	NF1-316	GATTGTTTGCACTAACCTGA	TTATTTTACTGGCCAAGCTG
chr17	29,562,845	29,563,017	NF1-317	TTATGTACAGAATGTGCAGG	TAACATGTTGCCAATCAGAG
chr17	29,576,014	29,576,211	NF1-318	AGAGAGCCTTGAGGAAAAC	GATCCTTCTTTGATAAAGCATTC
chr17	29,585,391	29,585,575	NF1-319	CATCGGTGCAGTAGGAA	TGAAAATTTAGTTGGAAGGGG
chr17	29,586,030	29,586,214	NF1-320	AACCCTGTTTTATTGTGTAGAT	TGGATTTATGTGAAACCGAAA
chr17	29,587,374	29,587,543	NF1-321	TGCATTTTTGAAGGTTTTTCC	GAAATCTTACCTGTTGCTGG
chr17	29,588,732	29,588,931	NF1-322	TCATAAAGCTGTTGGAAGAC	AACTCTCCTTCTCAACCAAA
chr17	29,592,246	29,592,445	NF1-323	GGCATCAGGTACATGAAAAA	AACTGGGAAAAACCAAACTT
chr17	29,652,958	29,653,141	NF1-324	TCGCTTTAAAACAGACTTTCT	CCCAGGACAGTCTATGAAA
chr17	29,652,985	29,653,184	NF1-325	GTGGTTTGTTGTTTTTCCTG	CAGGTAGTTTCTGTTGTTCA
chr17	29,654,717	29,654,900	NF1-326	TATCATTCATATCCGGACCC	AACAAACCCCAAATCAAACT
chr17	29,661,817	29,662,026	NF1-327	TCATTGACCATCACATGCTAA	TTGCTTGAATAGATGGGTACA
chr17	29,663,234	29,663,425	NF1-328	GAGGCCTCAGGTAAAATAGA	TTTTATTGATCCCAAGCCAC
chr17	29,663,764	29,663,934	NF1-329	ATGCTGTCCTTCAACAATTC	ACCACTAAAATGAAGCTGTG
chr17	29,664,385	29,664,558	NF1-330	GAAGAGACCAAGCAAGTTTT	TGTCAAAGCAAAAGTCTCTC
chr17	29,665,706	29,665,877	NF1-331	TCTGTTTTCCTAAAAGGCAC	ACATTCAACACTGATACCCA
chr17	29,667,507	29,667,696	NF1-332	ATATCTTCTTTGCCAGGACT	TAATCTAGGAACCTCAAGGC
chr17	29,676,164	29,676,337	NF1-333	GTTGCAAGAACAGTCAGAAT	TTGTACTTTTTGGGTGATCC
chr17	29,677,205	29,677,404	NF1-334	ACTTACAGTGTCTGAAGAAGT	GCCTCCATTAGTTGGAAAAT
chr17	29,679,270	29,679,440	NF1-335	TTCAGGACACTAAAGGAGAC	AAACTAACCAAGCAACTTCT
chr17	29,683,360	29,683,546	NF1-336	TCTTTGAGTCCTCAGTGAAA	CTGATTCCATTTCTTGCCTT
chr17	29,683,927	29,684,109	NF1-337	TGAAGTGATTATCCAGGTGT	CTAGAACAGTAAGAAGCAGC
chr17	29,684,202	29,684,380	NF1-338	ACAGTCTTCTACTTCTCACC	GAAAGACTTTGGGAAACACA
chr17	29,687,500	29,687,675	NF1-339	TACAGCAAACACAAATTCCA	GTTAAGGTTGGCAGTGATAC
chr22	29,999,999	30,000,194	NF2-340	CATCGCTTCCCGCAT	GTCTCTAGCTACACCCATAA
chr22	30,032,717	30,032,890	NF2-341	TCCCCATTGGTTTGTTATTG	CAGTTTCATCGAGTTCTAGC
chr22	30,035,052	30,035,221	NF2-342	TTTGTCTTTTGCTCTGCAAT	GGTAGCCTTGACTGATGTA
chr22	30,038,159	30,038,332	NF2-343	GAGTATCATGTCTCCCTTGT	TGATCCCATGACCCAAATTA
chr22	30,050,583	30,050,753	NF2-344	CTGTTCAGAAATGGCAGTTA	TCTGCTATGTCTTCCTGAAA
chr22	30,051,519	30,051,690	NF2-345	AGTGGCAAACAATACCAAAT	TAAACCAACAATGAATGGGC
chr22	30,054,133	30,054,305	NF2-346	CATCTCACTTAGCTCCAATG	CTCACTCAGTCTCTGTCTAC
chr22	30,057,166	30,057,354	NF2-347	ACAGTAGCTGTTCTTATTGGA	CCATCTGCAGTACACACA
chr22	30,060,955	30,061,147	NF2-348	CTGTGTTCTGCTTCATTCTT	TCACAAGATGTCACTCTGAT
chr22	30,064,266	30,064,459	NF2-349	AGGCAGTGAAGTAAATTTGT	CAGTTAAAACAAGGTTGTGC
chr22	30,067,788	30,067,987	NF2-350	ATGACTGTTTTTCTTCACCC	CAAGGAAGTCCCCAAGTAG
chr22	30,069,230	30,069,425	NF2-351	CTTCAGCTAAGAGCACTGT	CACCTTCTGCTCCATCA
chr22	30,069,311	30,069,510	NF2-352	GGAGGAGGCAAAACTTCT	CCTCCTCGCCAGTCT
chr22	30,070,801	30,070,980	NF2-353	TGACATCTCATCCTTTCCTT	AGAACATCACCAGGACTAAG
chr22	30,074,166	30,074,364	NF2-354	CGAAATTTCTCATTAACAGCC	TCACTCAGTCTAGTTCACAG
chr22	30,077,400	30,077,585	NF2-355	CTGATGCATGATACCCTCTT	TAATGGTATTGTGCTTGCTG
chr9	139,390,517	139,390,697	NOTCH1-356	CGCCGTTTACTTGAAGG	CAGCCACCAGCTACAG
chr9	139,390,641	139,390,838	NOTCH1-357	GGGGACTCAGGGGAC	GGCGGTGCACACTAT
chr9	139,390,779	139,390,957	NOTCH1-358	GAGGATGGCAGCGAC	CCACCACCACCACAG
chr9	139,390,894	139,391,112	NOTCH1-359	AGCTCCGGCCCAGGT	CCCAGATGATGAGCTACCAG
chr9	139,391,036	139,391,231	NOTCH1-360	CACCTGCTGGGTCTG	GAACCAATACAACCCTCTG
chr9	139,391,158	139,391,354	NOTCH1-361	CATGCTGCAGGGAGG	CTCTGGCACCAGCAC
chr9	139,393,285	139,393,474	NOTCH1-362	TTTGGCCCTCACTTCTC	CTACCCCATCTGCTTCTTT
chr9	139,393,593	139,393,763	NOTCH1-363	TGTGAGTTGATGAGGTCC	GCATCGGTGTACGTCT
chr9	139,395,110	139,395,307	NOTCH1-364	GCATCAGAGCGTGAGTA	CTCTGCAGATGGCTTCA
chr9	139,396,861	139,397,056	NOTCH1-365	GAAGAACAGAAGCACAAAGG	AGAGTGGGTGAGGAGG
chr9	139,397,621	139,397,804	NOTCH1-366	GGGCCACACTTACTCTG	CCCTCTCTGATTGTCCG
chr9	139,399,114	139,399,301	NOTCH1-367	GTCTCACTCACCCGC	GATGATCTTCCCCTACTACG
chr9	139,399,240	139,399,419	NOTCH1-368	CGGCACGCTTGATGG	TGGTGGTGGTGCTGATG
chr9	139,399,367	139,399,550	NOTCH1-369	GAAGTGGAAGGAGCTGTT	GTACGACCAGTACTGCAA
chr9	139,401,346	139,401,541	NOTCH1-370	GTTGTTAAAGCACTTGGGG	TCTCCCGAGTGTCCG
chr1	115,252,118	115,252,317	NRAS-371	ATGGATCACATCTCTACCAG	ATGTACCTATGGTGCTAGTG
chr1	115,256,456	115,256,649	NRAS-372	ATTGATGGCAAATACACAGA	AAAAATTGAACTTCCCTCCC
chr1	115,258,609	115,258,781	NRAS-373	GGTAAAGATGATCCGACAAG	ATGACTGAGTACAAACTGGT
chr4	55,129,755	55,129,952	PDGFRA-374	AGTGGGATAGTTTTTCTGGA	GTGTAAGGTTACAGGAGTCT
chr4	55,133,786	55,133,984	PDGFRA-375	ACATTTTGTTGTAGAGGTGC	GTAAGACACACACAAACCTC
chr4	55,139,716	55,139,915	PDGFRA-376	AACTTCCTGGACTATTTGG	GACTGTTGAGGAACTCACT
chr4	55,140,960	55,141,140	PDGFRA-377	CACTGGGACTTTGGTAATTC	CAAGCACTAGTCCATCTCTT
chr4	55,144,082	55,144,261	PDGFRA-378	AAAACAAGCTCTCATGTCTG	TGGAGAGTGGAGGATTTAAG
chr4	55,144,465	55,144,638	PDGFRA-379	ACAATTCATGGCTTTTCTGT	CTCTTTCTTTGGCTTCTCTG
chr4	55,151,599	55,151,782	PDGFRA-380	GTTGAGCTTCACCTATCAAG	AGGAGAGAGACAGGTAAGTA
chr4	55,151,988	55,152,178	PDGFRA-381	TTTCTTCCTTTTCCATGCAG	TAAAGTGAAGGAGGATGAGC
chr4	55,153,561	55,153,750	PDGFRA-382	AGACATGGGTTTAACTGTCT	GTTTCAGAACAGCCCAAATA
chr4	55,156,489	55,156,668	PDGFRA-383	AAAATTCACCTGGACTTCCT	AGGCAGAGGAATGATGTAG
chr4	55,161,230	55,161,419	PDGFRA-384	CCACAGTCTAGGTCTAGTTC	CCAGGTCTGAAGAGTCTATG
chr3	178,916,607	178,916,806	PIK3CA-385	CAGAACAATGCCTCCAC	CATCTTGAAGAAGTTGATGGA
chr3	178,916,707	178,916,902	PIK3CA-386	GTGACTTTAGAATGCCTCC	GTTGAAAAAGCCGAAGGT
chr3	178,916,783	178,916,976	PIK3CA-387	CCCTCCATCAACTTCTTCA	TTGTATCATACCAATTTCTCGAT
chr3	178,917,418	178,917,647	PIK3CA-388	AAACATGTTCATGCTGTGTATG	TGAAGATTCTACATTTGGAGGATAG
chr3	178,917,509	178,917,708	PIK3CA-389	TGATATGGTTAAAGATCCAGAAGT	GTAGATTAGTCATTTTCTTACCTTTATCT

Chr	Start	End	Amplicon-ID	Forward primers	Reverse primers
chr3	178,921,375	178,921,583	PIK3CA-390	TGTTGATGGCTAAAGAAAGC	CTTTACCTTATCAATGTCTCGAA
chr3	178,921,408	178,921,599	PIK3CA-391	TGCCAATGGACTGTTTTAC	AAGCATCAGCATTTGACTTT
chr3	178,922,192	178,922,387	PIK3CA-392	TGAACAAAAATTCCGTGGTT	TACTTCCTTACCTGGGATTG
chr3	178,927,300	178,927,492	PIK3CA-393	TCTTCGTGATTTGTAGGAGT	TTACCTCTTTAGCACCCTTT
chr3	178,927,942	178,928,119	PIK3CA-394	AGACTAGTGAATATTTTTCTTTGTTT	GGATTTGATCCAGTAACACC
chr3	178,935,988	178,936,157	PIK3CA-395	TATTTTACAGAGTAACAGACTAGC	AAAAAGAAACAGAGAATCTCCA
chr3	178,936,873	178,937,071	PIK3CA-396	AATGTAAGAAGTTTGGACTT	ATTTACCTGGGCTACTTCAT
chr3	178,937,647	178,937,831	PIK3CA-397	ACCAGTAATATCCACTTTCTTTC	CAAAAGAAAAAGTGCCCAAT
chr3	178,938,753	178,938,924	PIK3CA-398	AATTTTGCACGATTCTTTTAGAT	CTCCTGTTTGAGAATGTCAG
chr3	178,941,769	178,941,964	PIK3CA-399	TGTACAGTACTGAGGTTCTC	TAGTTGATGAGCAGGGTTTA
chr3	178,947,716	178,947,912	PIK3CA-400	CACCCTGTTTTCTTTTCTCA	TACTTGTCCATCGTCTTTCA
chr3	178,951,853	178,952,050	PIK3CA-401	ACTGACCAAACTGTTCTTATT	AGCCTCTTGCTCAGTTTTAT
chr3	178,951,991	178,952,185	PIK3CA-402	TTTGATGACATTGCATACATTC	CCAGAGTGAGCTTTCATTTT
chr10	89,624,185	89,624,359	PTEN-403	CCATCTCTCTCCTCCTTTTT	ACGTTCTAAGAGAGTGACAG
chr10	89,653,734	89,653,933	PTEN-404	TGATTGCTGCATATTTCAGA	TTCTAAATGAAAACACAACATGAA
chr10	89,685,231	89,685,402	PTEN-405	TGTTAATGGTGGCTTTTTGT	TCTACCTCACTCTAACAAGC
chr10	89,690,713	89,690,898	PTEN-406	AGATTCAGGCAATGTTTGTT	ACAGTCTATCGGGTTTAAGT
chr10	89,692,752	89,692,950	PTEN-407	GTTATCTTTTTACCACAGTTGC	AATTTGCCCCGATGTAATAA
chr10	89,692,846	89,693,044	PTEN-408	ATGGCTAAGTGAAGATGACA	GGAAGAGGAAAGGAAAAACA
chr10	89,711,859	89,712,057	PTEN-409	TTTTTCTGTCCACCAGGG	CTGTTCCAATACATGGAAGG
chr10	89,717,586	89,717,774	PTEN-410	ACTGGTATGTATTTAACCATGCAG	TTTTTAGCATCTTGTTCTGTTTGT
chr10	89,717,603	89,717,801	PTEN-411	CATGCAGATCCTCAGTTTG	CTCCCAATGAAAGTAAAGTACA
chr10	89,720,609	89,720,835	PTEN-412	TGTCATTTCATTTCTTTTTCTTTTC	TTTGCTTTGTCAAGATCATTTTT
chr10	89,720,706	89,720,905	PTEN-413	CCTCAGAAAAAGTAGAAAATGGAA	AACCCCCACAAAATGTTTAAT
chr10	89,725,022	89,725,220	PTEN-414	ATTTTAAATTTTCTTTCTCTAGGTGA	TGTAATTTGTGTATGCTGATCT
chr10	89,725,068	89,725,263	PTEN-415	GTAGAGGAGCCGTCAAAT	TCATGGTGTTTTATCCCTCT
chr13	48,878,008	48,878,199	RB1-416	GCTCCTCCACAGCTC	CTCTGCTCGCTCACC
chr13	48,881,416	48,881,615	RB1-417	GCTTGAGTTTGAAGAAACAG	TTTTGTATAGTGATTTGAAGTTGTT
chr13	48,916,727	48,916,896	RB1-418	GTTCCCAGGGAGGTTATATT	CGTTTCCTTTTATGGCAGAG
chr13	48,919,206	48,919,404	RB1-419	TCCTTTGTAGTGTCCATAAATTC	TCCCAGAATCTAATTGTGAAC
chr13	48,923,027	48,923,226	RB1-420	CTGGAAAACTTTCTTTCAGTG	TCCAAAGGAATGCCAATTTA
chr13	48,934,034	48,934,220	RB1-421	ACCATGCTGATAGTGATTGT	ATAGTCAAGGACACATAGCA
chr13	48,936,910	48,937,107	RB1-422	TGGATGTACAATTGTTCTTATCT	TGAAGTTAAATTACCTCATCTATATTAC
chr13	48,941,570	48,941,767	RB1-423	GTGTGCTGAGAGATGTAATG	AATCAATCAAATATACCATGTGC
chr13	48,942,511	48,942,710	RB1-424	AGCTGGGTCATCTATTTTCT	ACCTCTTCATCAAGGTTACT
chr13	48,947,451	48,947,636	RB1-425	ACATTTAACTTGGGAGATGG	TGGCTTACGTTAAAATAGGAAA
chr13	48,951,004	48,951,203	RB1-426	TCTGATTACACAGTATCCTCG	ATAGTACCACGAATTACAATGAA
chr13	48,953,711	48,953,902	RB1-427	TCTTTTTGTTTGTTTGTAGCG	GATGATCTTGATGCCTTGAC
chr13	48,954,180	48,954,378	RB1-428	CTGTTTCAGGAAGAAGAACG	CTGCTATATGTGGCCATTAC
chr13	48,954,193	48,954,402	RB1-429	AAGAACGATTATCCATTCAAAA	GTTTATTTATGAAAATTTAACTTACTGC
chr13	48,955,332	48,955,561	RB1-430	AGCTCAAGGGTTAATATTTCA	TTCCATGATTCGATGTTCAC
chr13	48,955,381	48,955,570	RB1-431	AGGAAGTACATCTCAGAATCTT	TGCAAGGGATTCCATGAT
chr13	49,027,011	49,027,210	RB1-432	CACTGTCAATTGTGCCTAAA	TAAGAGGACAAGCAGATTCA
chr13	49,030,302	49,030,511	RB1-433	CAACTTGAAATGAAGACTTTTCC	GCTCTTGAAAATAATCATCTACTAACC
chr13	49,030,300	49,030,521	RB1-434	GCCAACTTGAAATGAAGACTTT	CAGAGTCCATGCTCTTGAAAA
chr13	49,033,811	49,033,992	RB1-435	CTTATTCCCACAGTGTATCG	GGTGAAGTGCTTGATTTTCT
chr13	49,037,832	49,038,031	RB1-436	AATTCTGACTACTTTTACATCAATTT	ACCTATGTTATGTTATGGATATGG
chr13	49,039,127	49,039,347	RB1-437	TCCTCAGACATTCAAACGTG	TAGGGGGCTAGAGCAAAAAC
chr13	49,039,243	49,039,436	RB1-438	CCAGGGTAGGTCAAAAGTAT	TGAAATATAGATGTTCCCTCCA
chr13	49,039,343	49,039,525	RB1-439	CCCTACCTTGTCACCAATA	CCCTAAAGAGAAAACACACA
chr13	49,047,464	49,047,650	RB1-440	TTACTAATTGGTATTTCATCTTAACTT	CTATGCAATATGCCTGGATG
chr13	49,050,736	49,050,913	RB1-441	CTTTGCCTGATTTTTGACAC	CTTCCTTCAGCACTTCTTTT
chr13	49,051,401	49,051,599	RB1-442	GTAAGTCATCGAAAGCATCA	ACTTCTTGCAGTGTAAACAA
chr10	43,597,768	43,597,964	RET4-443	CAGACCTGACTTCTCTCTG	CTTGTCTCTGGGAAGCA
chr10	43,604,447	43,604,646	RET4-444	CTTGGTGGTCATTGTTGTG	AGAGGGAGTAGGTACTGG
chr10	43,608,931	43,609,128	RET4-445	ACTGCCCTGGAAATATGG	CTCACCCTGGATGTCTTC
chr10	43,609,864	43,610,058	RET4-446	CAGCCTGTACCCAGTG	GGAGATGGGTGGCTTG
chr10	43,612,011	43,612,183	RET4-447	CTTCCCTCATTTCCAACATAG	GTACCTTTCAGCATCTTCAC
chr10	43,613,711	43,613,905	RET4-448	CTCTCTGTCTGAACTTGGG	AATTTGATGACATGTGGGTG
chr10	43,615,513	43,615,703	RET4-449	CTATTTTTCCTCACAGCTCG	ATGGTGCACCTGGGA
chr10	43,617,297	43,617,472	RET4-450	TACTGAAAGCTCAGGGATAG	ACACTTACACATCACTTTGC
chr10	43,623,627	43,623,807	RET4-451	TGGGTTTCCAAGATATCCAA	ATGTGACGTTCACAAAGAAA
chr21	36,164,442	36,164,641	RUNX1-452	CTCCACACGGCCTCCT	GGCTCCTACCAGTTCTCCAT
chr21	36,164,567	36,164,755	RUNX1-453	GGAGGCGTTGGTGCAG	GCCACGCGCTACCAC
chr21	36,164,693	36,164,881	RUNX1-454	GCCTCCCTGCGCTTG	GACCCGCGCCAGTTC
chr21	36,171,579	36,171,769	RUNX1-455	GTGTTTTCAAGTGGCTTACT	TTCTCTTCAGATACAAGGCA
chr21	36,206,700	36,206,895	RUNX1-456	TACTTACCCTGCATCTGAC	ATCGGCAGAAACTAGATGAT
chr21	36,231,680	36,231,875	RUNX1-457	CCCAAGGAATCTGAGACAT	GGAAAAGCTTCACTCTGAC
chr21	36,252,839	36,253,030	RUNX1-458	TGACAGATAACGTACCTCTT	GTCCTTTGACTGGTGTTTAG
chr21	36,259,126	36,259,339	RUNX1-459	CTCCGGGCCAGTACCTT	AGATGAGCGAGGCGTTG
chr21	36,259,201	36,259,397	RUNX1-460	AAGTTGGGGCTGTCG	GTAGATGCCAGCACGA
chr18	48,573,468	48,573,652	SMAD4-461	AGCATTGTGCATAGTTTGAT	TCCAATGTTCTCTGTATGGT
chr18	48,573,481	48,573,676	SMAD4-462	GTTTGATGTGCCATAGACAA	ATAAGACTAACCTGAAGCCT
chr18	48,575,045	48,575,231	SMAD4-463	TATTTTTCTAGGTGGCTGGT	CCAATTCCAGGTGATACAAC
chr18	48,575,632	48,575,801	SMAD4-464	TGTTTCATTTGTTTTCCCCT	TTGTTAATGTTACTGCCTGC
chr18	48,581,156	48,581,351	SMAD4-465	TCAAGTATGATGGTGAAGGA	CAGGAATGTTGGGAAAGTTG
chr18	48,584,479	48,584,678	SMAD4-466	CTTCTTGTTCCTCTAGGTCA	CTTTTATAAAGGCTGCCTACT
chr18	48,584,574	48,584,763	SMAD4-467	GCAGAATGGATTTACTGGTC	GCAAATTAGGTGTGTATGGT
chr18	48,584,654	48,584,853	SMAD4-468	AAAAAGTAGGCAGCCTTTA	GCCCTTACAACACAAAAACAAGAG
chr18	48,586,210	48,586,394	SMAD4-469	TGGAATTTTTGTTGTCTTTTCTT	TACTCATCTGAGAAGTGACC
chr18	48,591,780	48,591,979	SMAD4-470	TTATTTCCTATAGCTCCTGAGT	TACCTTGCTCTCTCAATGG
chr18	48,591,805	48,591,999	SMAD4-471	GGTGTTCCATTGCTTACTTT	CTATCTGACTATACAATCAATACCT
chr18	48,593,376	48,593,563	SMAD4-472	TTTTCTTCCTAAGGTTGCAC	ACTAACCTTTATATATGCACTTGG
chr18	48,593,386	48,593,589	SMAD4-473	AAGGTTGCACATAGGCAAAG	GTCTAAAATATTCAAATAAAATTGTAACTAACC
chr18	48,602,941	48,603,170	SMAD4-474	GCATTGGTTTTTAATGTATGGAA	AAAAAGAATGAAAAGCATACTTAC
chr18	48,604,617	48,604,813	SMAD4-475	TTCTGTTAGGTCTGTCAGC	AATCGGCATGGTATGAAGTA
chr18	48,604,667	48,604,849	SMAD4-476	CGCTTATGCATACTCAGGA	CGGTAAAAGACCTCAGTCTA
chr7	128,828,883	128,829,056	SMO-477	CACAGGTCGCCTGAGC	GCAGCAGCAGCAGCA
chr7	128,829,083	128,829,270	SMO-478	GCCTCGAGCGGGAAC	GCCAGCAGTGTGGAGGT
chr7	128,844,930	128,845,120	SMO-479	GGTCCTGGAAGTGTATCTC	CTCTTGGGGTTGTCTGT
chr7	128,846,015	128,846,195	SMO-480	CATCATCTTTGTCATCGTGT	GATTGCCACAGTGAGGA
chr7	128,846,347	128,846,545	SMO-481	AAGAACTACCGATACCGTG	TATAGGAGCTAGCTGGGG
chr7	128,849,170	128,849,369	SMO-482	CTTCAGCTGCCACTTCTA	GACACCATCCATTGAATCTG
chr7	128,850,263	128,850,462	SMO-483	TCAAGAATCGCCCGAG	CAAAACCTAAAGATGGGGTC
chr7	128,850,746	128,850,933	SMO-484	CAAGATTTGATGGGAAGTGG	CAGTGTGCATGCTGAAG
chr7	128,851,432	128,851,626	SMO-485	GGGAAGTCACTATTCCTTCT	CTTGAACTCTCATACCTGGA
chr7	128,851,779	128,851,960	SMO-486	ACTAACAGGTTAAGTGCTCC	TCTTCCTCCTCTTCTTCTTC
chr7	128,852,160	128,852,340	SMO-487	TCAGGATCCATTTCTGCC	GAAGGTATTGGTTCCTCTCT

Chr	Start	End	Amplicon-ID	Forward primers	Reverse primers
chr15	73,564,854	73,565,043	SNP1-488	CATAATTGTGAATTGGCAGC	CATCATGACTCACACCTTAC
chr19	9,968,050	9,968,237	SNP10-489	AGAGATTCCTCACCTCCT	TTATGAGTCAGAGGTTGGAG
chr19	55,107,643	55,107,814	SNP100-490	CTTCTCTCCAGGACAGTTC	GTGTATTGATCTGAGACGGA
chr22	24,621,996	24,622,195	SNP101-491	TAGGAGAGGAGGAGTGAAG	CTCATCCGCCAACAGAT
chr10	91,143,313	91,143,490	SNP102-492	AATTCAGAAAGAACATGCCA	CTCTGGACACTCCATTCTAT
chr5	108,233,367	108,233,542	SNP103-493	CTTCTGCTAAGCCAAAAACA	GTAACTGATCGTGCATCTTC
chr11	112,104,188	112,104,369	SNP104-494	CAACCTCCAGAAAGTTCTTC	TCACGTGTTGACCTCTTAAT
chr11	71,712,418	71,712,617	SNP105-495	CTTCTCATGACCTTTCCTTC	CTCTCCTTGGCTCCT
chr3	45,754,583	45,754,777	SNP106-496	TAAGCTTGGCAGGAGATATT	TCTTGGTGATTAGGAAGTCA
chr2	33,487,785	33,487,957	SNP107-497	CCAGGAAATACCTTCATTGG	ACAAAAATTTCCCGTCTTACA
chr4	72,622,480	72,622,673	SNP108-498	AACTCTACATCTGGAAGCTC	TCAGGAAACTAACAATGCAC
chr11	62,009,666	62,009,862	SNP109-499	GCTAGACTCCCTAGGTACA	GAGCTAGACTCATGACTGAT
chr12	13,764,703	13,764,898	SNP11-500	TACTCAAAGACAAAGACAGC	CAACACATTGTGGTTTGTTC
chr2	26,698,923	26,699,095	SNP110-501	CCTGAGGTTCCAGGATG	AAGGAGTTCCTGTGCG
chr14	62,213,524	62,213,702	SNP111-502	TGCCAGGTAAACTAAAAACC	GTGGTAATCCACTTTCATCC
chr10	123,970,053	123,970,236	SNP112-503	TGGACTTTGACAACATTGAG	GAAGAAGCACCATCAAAGTC
chr5	180,166,267	180,166,436	SNP113-504	ATTGTGGATGGATTGGAGAT	TTCCTGCAGCACTTATTCTA
chr10	123,970,445	123,970,618	SNP114-505	CTAGCATCTGAGACGAAAAC	TGAGTTCTGCACTCTGC
chr6	26,385,133	26,385,306	SNP115-506	CTTTCATCCCTGGAGTTTTT	AATGTAGTGTTTTCTCCCAC
chr4	3,009,368	3,009,562	SNP116-507	TTTCCCATATTCAGTGCCTA	TACATACCTTTCCAGCCATT
chr19	55,098,603	55,098,772	SNP117-508	GGTGGACATACAGGAGTC	GAGCCTCAAATAGCAGAATC
chr1	42,865,236	42,865,433	SNP118-509	ATCAACAAATTCTGGACGTT	AGATTTCAGATAGGCCAAGA
chr22	32,587,211	32,587,384	SNP119-510	AAACGGACACGTCAAATC	TTGGTTTCTGTCCATTTGC
chr3	43,389,208	43,389,397	SNP120-511	TCAACCAGATCTTTGAGGAA	TCAGAATCATCATCACTGGT
chr8	7,308,614	7,308,794	SNP121-512	AGGATGATAGGGTGTGTTTT	TTTCCTTAGAGACCAAGACC
chr12	12,332,714	12,332,890	SNP122-513	GGCTTGAAAATGAAAAAGCA	GAAATTCCGAAAATTGAGCG
chr1	152,282,078	152,282,258	SNP123-514	AACGTCCAGACCTTTCC	GATTCATCTGTAGTCGGAGA
chr19	814,414	814,607	SNP124-515	CATCAGAACCTGCCACT	ATGTACTTCAACTCGGTCAT
chr15	75,668,102	75,668,291	SNP125-516	CAGCTTATCCAGACTATCCA	AAAGAAAACTCACTTTGGGG
chr11	26,700,270	26,700,447	SNP126-517	CTGATGATTACTCCAGCAAC	CCCTAAAATGAATCCTGAGC
chr19	10,127,758	10,127,943	SNP127-518	GAAGAAGGAGACACTGGAG	CTATACTCAGACCAGGATGG
chr15	50,399,033	50,399,217	SNP128-519	CTAGACCTGTGTGCTTTTTA	TCTTTTTCTTAGGTAGGCCA
chr2	165,754,871	165,755,043	SNP129-520	AATACAGACTAAAGCAAGCG	CTGCTTTCCTGACAATTTCT
chr11	5,529,104	5,529,296	SNP13-521	CTAGACTCTATACCTCCTGC	AGGATGAGATACAACCACAG
chr3	38,057,924	38,058,122	SNP130-522	GATAACCACAGCCAGTGT	CTTCTCCATTGTCTTCAAGG
chr17	39,975,429	39,975,627	SNP131-523	AATTTTATGGTTCAAGCCCT	ATCAAGGAGCCATTGTAGT
chr17	1,631,520	1,631,699	SNP132-524	CTATGTGACTGAGTCTCCC	CTTTGCTTCAAGTCCTGG
chr1	152,285,047	152,285,228	SNP133-525	TGATGGTGACCAGCCT	CACCAGCTCCAGTCAG
chr9	35,661,031	35,661,224	SNP134-526	CACAGAGACATGGAACCTA	ACAACCAACTAGGATCGAAA
chr18	50,278,553	50,278,723	SNP135-527	GGAAGAAAGATGGCATTCAT	TCCGACTAATAATTGAGCCA
chr10	88,659,635	88,659,831	SNP136-528	CCTGTTGTCATAGGTAGGTT	TAGAAATGAGCAAAACCAGC
chr21	31,913,952	31,914,150	SNP137-529	AGAGTATGAGAATCAGCAAGT	GAGATACTATGGCAGCTACT
chr18	52,258,509	52,258,707	SNP138-530	CAAGAATGGACAGAAAGCAT	GGGTTGCTCTTTATTTTGGA
chr7	50,514,501	50,514,686	SNP14-531	GAGTCACTCTCTTGAGAACT	GCAGTCTGGATTGTCAATAA
chr12	46,321,602	46,321,796	SNP140-532	TAACAGATTGTCTGTCCACT	CTGTATCTTGTCCCCTAAGT
chr1	152,283,683	152,283,852	SNP141-533	CTGATTGTTTGTCCTTACGA	GTCAAGGAGAGGAGGAAG
chr5	135,228,080	135,228,254	SNP142-534	TCATATCTTGCCTCTCATCC	GCTTGGCTCTACATCATTTG
chr11	59,860,812	59,860,983	SNP143-535	AGGTTTATTGAATGTGCCAG	ACTGTGGATGTGGATATAGG
chr5	147,805,013	147,805,185	SNP144-536	CTGGAAACAGTACTTTGCAT	CCAGACTTCTTGGAGTTCTA
chr17	4,918,051	4,918,246	SNP145-537	GATATGGACATCAAGCTGAC	CTCTTTATATGGCTGCACTC
chr17	5,264,814	5,265,001	SNP146-538	CAAAGATCAGGAAAGAGCAA	CAAGAGGAAAAACGTGAGAA
chr3	52,130,581	52,130,766	SNP147-539	CTACCTGAGTGAGGACATC	CTGGATAGACTGAGGAACTT
chr18	61,564,863	61,565,040	SNP148-540	GGTCTCCTAATTTCAATGGG	CTAGCTTCTTCTGCACATTC
chr8	87,641,145	87,641,333	SNP149-541	ACCTAGCATTCTTTGAGAGT	GTGGAGTGACCATATTTGTG
chr12	77,419,480	77,419,668	SNP15-542	GTGGACGACTTTGGATTAAC	CATGGTCTTACCATCTCCA
chr12	131,623,697	131,623,876	SNP150-543	CCTTTATGCTTTGCAGATGA	GGGGCATTTCATTCTGTTT
chr7	99,526,652	99,526,847	SNP151-544	CTCCAAACATGGTCTTTAGG	CTGCTCTGGGCTTATGT
chr2	202,264,097	202,264,278	SNP152-545	TACTTGATCAAAGGCTTGTC	TTCTGAACTGCCATTTAACC
chr19	55,494,560	55,494,729	SNP153-546	CTACTCCTTCATCCACCTC	AGCCTGCTTGGATCAG
chr2	61,468,600	61,468,797	SNP154-547	TATCAACCTGTAGAACCTGT	CTTAAAGACACACAGTGCAT
chr15	75,982,832	75,983,025	SNP155-548	AGCCCTCATGCACATC	GTTTCTGAACGCCTCCT
chr1	53,279,331	53,279,529	SNP156-549	GCCCATTTAATATCCAGAGG	GGAAGACAAAAGACACACAA
chr11	6,942,675	6,942,871	SNP157-550	ACTCTACCATCATGACACAA	CATTTCTGTGCTGTAAGTGT
chr2	242,192,788	242,192,958	SNP158-551	CATTCCAAGGGGTACCTTA	GCTGTGCCATTCTGTTTTA
chr21	46,057,520	46,057,711	SNP159-552	CCAATCAGGCTACACCA	CATGAAGGGGAAGACTCG
chr19	731,092	731,262	SNP16-553	CAGGTTGGAGAAGGAAATTG	CCCAGTCACCCCCTTA
chr6	168,352,130	168,352,325	SNP160-554	CCAATCCGAACAGACCT	CCTTCTCCTTCTCATACCAA
chr5	132,198,134	132,198,320	SNP17-555	GCAGAGGAAAAAGAAACTGA	AATTCTTTGACTTGACTGCC
chr16	4,849,569	4,849,755	SNP18-556	AGGTAAGTGCTTACAGGTT	CAAGCCATTTACCTGCTTAC
chr1	46,500,467	46,500,666	SNP19-557	ACAAAGCTTCACTTGTCAC	GGCAGTTCCTTCTTTAGTTC
chr16	58,616,911	58,617,089	SNP2-558	AGAGGACAGGACATTAACTC	CAGTATTGAAGAATGTCGCA
chr11	5,364,385	5,364,580	SNP20-559	TGGCAATAAAACGGTCATAG	TTCTTTCTGGCCATGCT
chr19	39,906,997	39,907,184	SNP21-560	GGAGGACTTGGAGAACAG	GTGACATGGGAGTCAGG
chr17	5,485,346	5,485,527	SNP22-561	CTTGGAAGAGCTTGGTAGA	AAAATTGCCCCTCTACTTCA
chr11	117,096,511	117,096,688	SNP23-562	GGCAATGGACTTATTAGCTG	GTTCAACAAGCACTATCAGA
chr1	70,819,916	70,820,091	SNP24-563	TGGTGATGGTTACTGAAGAT	ATGACCGGGGAGAAGAT
chr9	100,070,268	100,070,455	SNP25-564	TTGGAATTGAGACACCAAAG	GAAGGGGGATGGAGAAAA
chr6	39,563,818	39,564,005	SNP26-565	TGCCCATTTTACCTGTTCTA	ACTGCATTTTCACCATTCAT
chr7	796,290	796,483	SNP27-566	ACTCACTCTGCCTAACAC	AAGATCAGCTTCAGAAACAC
chr3	8,775,472	8,775,661	SNP28-567	GACACTGAATTGGTCTCTCT	GTTAATGTTCTTGGGGTCTC
chr5	137,625,158	137,625,348	SNP29-568	TAACCAGTTACCATCTCCAG	TAAGGGTCACTTGGAAAGAA
chr19	43,762,958	43,763,144	SNP3-569	GTTTGGACTTAAGCTGGTG	GGGGTAAAGCGACCC
chr11	18,291,268	18,291,442	SNP30-570	GCCAGAGAGAATATCCAGAG	CTCACAGCCAGATCTCC
chr8	144,620,463	144,620,639	SNP31-571	CTCTGGGTCAGCTATGAG	ACCTGTCGAACTAACAAGT
chr3	56,763,532	56,763,725	SNP32-572	TTCTTTGGCTTGACGAATAC	CACTACGTGTGTTCTCTTTG
chr3	56,766,410	56,766,597	SNP33-573	GAGTGATCACAAGCACTTC	GTTCTCCTGGAGGTAGATTT
chr12	121,436,942	121,437,140	SNP34-574	TGGATCTCCAACTGCTG	ATGAGCATAGTCTGCGG
chr2	175,263,010	175,263,179	SNP35-575	AAATGGAACCTTTTTCCTGT	TTCACCTACCTTAAGACGTT
chr14	74,424,787	74,424,985	SNP36-576	CTTTTTCCTCAGCCTATGTG	ACTACCAACAATTTGGTCTG
chr4	88,732,806	88,732,998	SNP37-577	GTGGGTTTGAACCTACAAC	TACTCATCTTCATAGGCTCG
chr19	39,908,496	39,908,689	SNP38-578	TACCCATGCTTGTCTCTG	TCCGAGAGAACAGGAAGA
chr19	38,160,627	38,160,803	SNP39-579	CATCAGTGTGAATTTTCCGA	CACACTAGTGAGAAACCCTA
chr14	21,109,648	21,109,831	SNP4-580	AGGTTACCCAGAAAGAAGTA	ACAGTAGTGATCCAACAGAG
chr2	21,263,798	21,263,993	SNP40-581	AACATGACTTACCTGGACAT	ATAGACAACCTCAATGCTCT
chr1	248,224,678	248,224,853	SNP41-582	GGAGGAAGGAAAGCCTACC	GCTATAGATGATGGGGTTGA
chr14	102,901,059	102,901,255	SNP42-583	CCAAAGCACTTTTTGTGAAG	CAGCATGCCAGAGATTTG
chr6	17,675,187	17,675,382	SNP43-584	ACCGTATGTTGTTTTTCCAG	GACAAGAGTCTTTTGCTGTT
chr1	200,376,589	200,376,760	SNP44-585	AATAAAGACCTGGCTGAGAT	TTTGTCAGTTTGTCTTCACC

Chr	Start	End	Amplicon-ID	Forward primers	Reverse primers
chr18	44,561,525	44,561,694	SNP45-586	CAAGGCGGAGAGTTTCT	CAGCCCCAAAGACAGAG
chr1	22,919,931	22,920,117	SNP46-587	GTACTACGAGAAGGTACCAC	GCTCGGACCTGGAAC
chr17	29,623,204	29,623,381	SNP47-588	AATGTAGACAAGTTTCTGCC	AGAAGGGTGTATCTGTTTGT
chr2	120,005,437	120,005,636	SNP48-589	CTTCTATGCCTACAACTTCG	GGAAGCGCTGGTACTT
chr17	72,937,664	72,937,835	SNP49-590	CAGAAGGCTGGACAACT	CCACATAGTAGAGAAGCCAA
chr1	17,396,521	17,396,691	SNP5-591	CCTTCTGGATCTGGCTG	TGACATCCTCAAGAAGGAG
chr5	102,894,549	102,894,723	SNP50-592	TTACCAATGAAAAGCAGCAT	GAGAGGAGGAAGATGGATTG
chr5	31,538,553	31,538,741	SNP51-593	AAACACTGCCTCTACTAACT	TCTAAAACAAATGCCTTCCC
chr6	76,425,218	76,425,410	SNP52-594	AGCAAAGAGAAAAGAAAGCA	CAAATCACTGAGTAGTGAGC
chr14	91,636,358	91,636,556	SNP53-595	CCCAGTTCTGGAAATACGA	TGTGTCCCTACCTTGTATG
chr8	22,021,366	22,021,550	SNP54-596	TGGGATAGAAACTCACTTCC	ATGTAGTAGAGCGGCAC
chr11	6,898,371	6,898,565	SNP55-597	TTCAGTTTCCCTTTTTGTGG	CATCCTGAAGATAGTGGAGA
chr4	1,729,891	1,730,086	SNP56-598	AATCCCTAAGGAAGCCTG	CTGAGGACTCAGGGTCA
chr3	49,455,251	49,455,426	SNP57-599	CAATCTTGGTACCCTCCAT	TCTCATGTGTCATTCTCCAG
chr3	129,155,624	129,155,805	SNP58-600	ACAGTTCTATCAAGCTGACT	GTTAGAAAGCCCAAAGGAAA
chr3	49,689,151	49,689,328	SNP59-601	CCTCCGAGATCCACAAG	ATATCCTCCAGCTCCTCAT
chr12	132,631,714	132,631,900	SNP6-602	GCCCTTTTCTCAGCTCT	TTCCCACTTGGACTTCTC
chr19	35,836,400	35,836,584	SNP60-603	AGGGGATAAAATGTCCTCTG	GTGTAGCTAATGCCATCTTC
chr2	26,502,813	26,502,983	SNP61-604	TATAAGGCTTGTGGTTCCAT	CAAGAAAGAAGAATTTGCAGC
chr20	62,200,531	62,200,729	SNP62-605	TCCTCATAGAGAAACTGGTG	ACCCTGAGGAGATGGAG
chr3	49,362,256	49,362,450	SNP63-606	AGAAGCAAAAAGTATCTGGC	GAAGCTATTCAACATCCCTG
chr10	17,875,726	17,875,917	SNP64-607	CCAGATAAACTCCAAATCCG	CTTGTGCTGTTGACTTCTAA
chr15	74,336,457	74,336,654	SNP65-608	GTGAGGTCTCTAGATGGTG	CCTTGGAGTAGATGCTGA
chr3	49,714,178	49,714,357	SNP66-609	CTTGAGACTGAGTGTCTGT	AGGGAGTCATCCGAGAG
chr17	35,346,572	35,346,760	SNP67-610	CTTGACTCAGATCGACCATA	GCCTCATAGAAACTATTCCC
chr3	49,721,378	49,721,561	SNP68-611	TGTACAGGCATAAAGAGGAA	TAATCCCCAACCGAGTATG
chr2	26,455,017	26,455,216	SNP69-612	GTTAGACTCACCATTTTGGG	CCTTATCCATCTCAAGTTGC
chr1	145,533,056	145,533,251	SNP7-613	ATGGGATTCTTTTTGGGATG	GATGTATTTCCTCCCCTACC
chr19	36,303,631	36,303,814	SNP70-614	CCATCAAAGCTCAGGGA	CTTTGCCCAGTTAATCAGTC
chr3	48,725,532	48,725,730	SNP71-615	AATAAAGACTCCAAGCACAG	GATAAGTGAGGAGAGTGGG
chr19	21,131,514	21,131,689	SNP72-616	GCAAGTGGAGTAATTTGTCA	CTCATCCATACTTTCACAGC
chr3	49,156,350	49,156,525	SNP73-617	AGGACAGAGAGAAGAACAAG	GGACACCACTAGTAAGAAGA
chr9	2,029,095	2,029,278	SNP74-618	CCAATTCTTGGGCCTAGT	GTTGAGTTTGAATGGGAGAA
chr6	146,007,305	146,007,502	SNP75-619	TGTTGTGTGCTTCATTTCAT	CAGCTGCTTTGAGGATATTT
chr20	62,196,042	62,196,224	SNP76-620	AAGCTGGCCACATCAG	CAAGGTGCTGCAGAAATAC
chr12	53,708,773	53,708,951	SNP77-621	ATGGTGAGCAATCAGAAAAG	TGTCCTGTGTCTATCTTGTT
chr19	54,849,346	54,849,519	SNP78-622	GTTGTGTTGGTGTGAGTTC	TTTCTCTTGAAAGCCTACAG
chr4	95,186,020	95,186,201	SNP79-623	GGATGGAACATAGAACAACC	CCACTTATGTGCCATTACAG
chr11	118,177,952	118,178,146	SNP8-624	CAGGTACCCACAACATTTAC	CAGAAACTCCTTTTCCTGAC
chr9	86,278,821	86,279,001	SNP80-625	ATCTGCTGAATAAACTGCTG	TTTTGCAGGACCTTAGGATA
chr17	1,703,930	1,704,099	SNP81-626	ATATCTTTTGTGGCAACGAG	AAGATGCAAGAAAAGGACAG
chr5	140,603,186	140,603,356	SNP82-627	GAGGAAACAGAAATTGGCTC	GTCTCGGTCTAGTTTCTCAT
chr7	76,922,359	76,922,547	SNP83-628	GAACTCCAAAAGGAAGTCAG	TAGAAATGCAATACAGGAGC
chr11	71,717,202	71,717,374	SNP84-629	TAGAGACTGAGTAGAGGAGG	GGAATGGGAAGCTTCTGTA
chr7	76,132,768	76,132,954	SNP85-630	GCAAAACCATCTATGGAGAG	GGGTGGGAAGAGGAGT
chr2	113,737,496	113,737,681	SNP86-631	AGATCCACTTGAGGATACTG	AACACATTTCTGGATTCTGG
chr5	140,605,016	140,605,186	SNP87-632	AATGGCGAGCCTCCT	GAAGAGGAAGAGCGACG
chr5	108,233,522	108,233,712	SNP88-633	AGAAGATGCACGATCAGTTA	GAGGTGACTGACAAGTCTAT
chr10	91,143,058	91,143,230	SNP89-634	TGCTGCCTATTTTTACAGTG	TTCCCACATTGTATTTGGTG
chr14	64,686,104	64,686,276	SNP9-635	AGAACTGAGAGTGAAGAGAC	TGGAACTGAAATGGAAGAGA
chr18	8,784,670	8,784,845	SNP90-636	GGCTTTCAAGAAAGAGCTG	TTACCTGCAAGTCTGGG
chr11	5,221,319	5,221,516	SNP91-637	ATTGAATCGGATGTCTGAAC	CTACGTTATTCCTCCATCCT
chr6	43,582,016	43,582,192	SNP92-638	CCAAATCCAAGTCTTCTAGC	TTGATGAGATACGGCAGAAA
chr5	180,166,743	180,166,942	SNP93-639	CTAGGTAGATGAAGAGCTGA	CTTTGGCAACACCATCATC
chr19	11,227,506	11,227,705	SNP94-640	GACCTCTCCTTATCCACTTG	TAAGTGCTTGCATCTCGTA
chr17	10,543,362	10,543,548	SNP95-641	AGTTTTTCCTCTAGCTCCTT	AGTACAACATTCGCTCATTC
chr14	50,074,139	50,074,330	SNP96-642	GGTTTCCTTTCAGCTATGAG	CCCACAGTAAGAAGTCTGAA
chr10	100,011,350	100,011,546	SNP97-643	ATCTGTGATATCCACCCAC	ATGATGACACTGAACTGGTA
chr9	19,058,345	19,058,540	SNP98-644	ATAGGACTATTTGGCTCCAG	CTTGTAACAAACCTTCCACA
chr3	171,969,164	171,969,361	SNP99-645	GCCCTCCTTCTTCTATCTAC	TCACTCTATGGCAAACATCT
chr20	36,031,722	36,031,898	SRC-646	CTTCCTGGAGGACTACTTC	AATTCAGCCCCACAGAG
chr19	1,206,888	1,207,072	STK11-647	GGGACTCCAGGACCC	GGTCCCCCATCAGGTA
chr19	1,207,008	1,207,207	STK11-648	CGAGGTCATCTACCAGC	CTTACTTCTTCACGTTGGC
chr19	1,218,401	1,218,599	STK11-649	TCCTCTCTGTCCCAGG	CTTCAAGGAGACGGGAAG
chr19	1,219,320	1,219,508	STK11-650	CACGTATATGGTGATGGAGT	GAATATCAGGACAAGCAGTG
chr19	1,220,349	1,220,564	STK11-651	GGACGGGTGTGTGCTG	GCAGCCCTCAGGGAGT
chr19	1,220,447	1,220,630	STK11-652	GAACCTGCTGCTCACCAC	GGAGCCCTGGCTGGT
chr19	1,220,549	1,220,725	STK11-653	ACTCCCTGAGGGCTG	GGCACTTACAGGGTGAC
chr19	1,221,186	1,221,358	STK11-654	CTTTCTTCCCTCCCCTC	AGAGGGATGAGGCTCC
chr19	1,221,890	1,222,073	STK11-655	GTTGGTGGGGTCTCAG	CTCAACCAGCTGCCC
chr19	1,222,964	1,223,163	STK11-656	CTGCTTCTGGGCGTT	GTGAAGTCCTGAGTGTAGAT
chr19	1,222,987	1,223,186	STK11-657	GTTCCGGAAGAAACATCCTC	CCCCGCCAGACTCAC
chr19	1,226,515	1,226,705	STK11-658	TGTATGAACGGCACAGAG	GCGTTGTCCCCACAT
chr17	7,572,849	7,573,048	TP53-659	AGACCCAAAACCCAAAATG	ATGTGATGTCATCTCTCCTC
chr17	7,573,860	7,574,046	TP53-660	GAATCCTATGGCTTTCCAAC	TCTGTTGCTGCAGATCC
chr17	7,576,820	7,577,017	TP53-661	TTCCACTTGATAAGAGGTCC	TAAGCAAGCAGGACAAGAA
chr17	7,576,898	7,577,092	TP53-662	GGGAGAGGAGCTGGT	GCGCACAGAGGAAGAG
chr17	7,576,997	7,577,181	TP53-663	GCTTCTTGTCCTGCTTG	TGCTTCTCTTTTCCTATCCT
chr17	7,577,443	7,577,623	TP53-664	CAAGCAGAGGCTGGG	TGTGTTATCTCCTAGGTTGG
chr17	7,578,151	7,578,347	TP53-665	CCAGAGACCCCAGTTG	CGACAGGGCTGGTTG
chr17	7,578,259	7,578,455	TP53-666	ACTCGGATAAGATGCTGAG	GCCATGGCCATCTACAA
chr17	7,578,383	7,578,581	TP53-667	AGCAGCGCTCATGGTG	AACTCTGTCTCCTTCCTCTTCC
chr17	7,579,296	7,579,494	TP53-668	CCTCAGGGCAACTGAC	AGAATGCCAGAGGCTG
chr17	7,579,416	7,579,615	TP53-669	AGGAGGGGGCTGGTG	GACTGCTCTTTTCACCCATC
chr17	7,579,713	7,579,901	TP53-670	TCAGGAAGTCTGAAAGACAA	GCAGTCAGATCCTAGCG
chr3	10,183,510	10,183,698	VHL-671	GGTGGTCTGGATCGC	GCCTCCATCTCCTCCT
chr3	10,183,596	10,183,794	VHL-672	AGTACGGCCCTGAAGAAGAC	CATACGGGCAGCACGA
chr3	10,183,716	10,183,915	VHL-673	TGCTGCGCTCGGTGA	TGCTATCGTCCCTGCTG
chr3	10,188,164	10,188,362	VHL-674	GTGGCTCTTTAACAACCTTT	ACCACAACAACCTTATCTTTT
chr3	10,191,441	10,191,639	VHL-675	CCACTGAGGATTTGGTTTTT	ATCCGTTGATGTGCAATG
chr3	10,191,489	10,191,688	VHL-676	GATGCCTCCAGGTTGTCC	AAAGCTGAGATGAAACAGTGTAAGT

TableS3 : Sequencing and mapping metrics

run	sample	read	total reads	mapped		mapped without gap	
			No.	No.	%	No.	%
run1	Breast_Blood	fwd	42,550,041	24,966,884	59	24,648,440	58
		rev	42,550,041	21,263,067	50	21,081,411	50
	Colon_Blood	fwd	37,739,948	22,816,056	60	22,533,562	60
		rev	37,739,948	20,655,094	55	20,473,239	54
	Ovarian_Blood	fwd	36,109,150	22,183,665	61	21,931,730	61
		rev	36,109,150	20,014,816	55	19,852,461	55
	Colon_Xenograft	fwd	36,112,189	21,969,131	61	21,581,453	60
		rev	36,112,189	20,040,263	55	19,810,298	55
	Ovarian_Xenograft	fwd	33,976,079	20,696,364	61	20,471,385	60
		rev	33,976,079	19,414,659	57	19,251,611	57
	CAL-A	fwd	30,860,030	19,126,216	62	18,901,099	61
		rev	30,860,030	18,071,929	59	17,918,595	58
run2	Breast_Xenograft	fwd	30,311,000	18,669,098	62	18,362,370	61
		rev	30,311,000	19,391,473	64	19,214,321	63
	Sarcoma_Xenograft	fwd	32,732,546	19,595,780	60	19,330,845	59
		rev	32,732,546	18,966,337	58	18,807,292	57
	CAL-B	fwd	35,283,984	21,044,008	60	20,716,982	59
		rev	35,283,984	20,378,236	58	20,210,154	57
	CAL-C replicate 1	fwd	30,059,779	18,978,908	63	18,697,006	62
		rev	30,059,779	18,359,175	61	18,224,396	61
	CAL-D	fwd	36,088,546	21,511,991	60	21,183,761	59
		rev	36,088,546	20,805,319	58	20,639,342	57
	Sarcoma_Blood	fwd	42,015,874	22,903,178	55	22,549,978	54
		rev	42,015,874	20,203,874	48	20,027,166	48
run3	Breast Primary	fwd	35,596,030	17,784,222	50	17,469,784	49
		rev	35,596,030	23,987,344	67	23,766,069	67
	Colon Primary	fwd	39,978,058	15,733,811	39	15,403,527	39
		rev	39,978,058	20,199,144	51	19,882,799	50
	Breast Xenograft WGA	fwd	38,537,327	17,865,528	46	17,444,720	45
		rev	38,537,327	22,884,846	59	22,574,743	59
	CAL-B_WGA1	fwd	36,739,465	17,934,516	49	17,539,297	48
		rev	36,739,465	25,929,075	71	25,693,018	70
	CAL-B_WGA2	fwd	38,757,101	18,520,810	48	18,103,898	47
		rev	38,757,101	24,887,211	64	24,617,780	64
	CAL-C replicate 2	fwd	33,971,192	21,548,712	63	21,252,446	63
		rev	33,971,192	19,499,951	57	19,303,106	57

TableS4a: Base Pair coverage statistics

Sample	mean coverage	number of base pairs with coverage:					
		>=0	=0x	>1000x	>5000x	>10,000x	>50,000x
CAL-A	24,914	164,944	475	159,728	154,344	147,235	117
CAL-B	26,059	164,944	1,604	154,173	143,701	132,321	2,232
CAL-C replicate 1	23,843	164,944	1,250	156,535	147,196	136,154	100
CAL-C replicate 2	23,488	164,944	696	156,687	145,049	130,721	1,174
CAL-D	26,617	164,944	1,589	154,117	143,779	131,933	3,042
CAL-B WGA1	22,674	164,944	626	158,121	146,525	124,297	6,440
CAL-B WGA2	20,548	164,944	593	157,822	145,797	123,561	4,066
Breast_Blood	25,044	164,944	1,257	154,152	140,016	126,657	7,949
Breast_Xenograft	24,005	164,944	851	158,212	149,851	139,161	282
Breast Xenograft WGA	17,396	164,944	1,055	154,650	137,451	108,056	3,656
Breast Primary	21,696	164,944	1,350	154,189	144,462	128,235	3,043
Colon_Blood	27,572	164,944	1,133	155,911	147,433	137,705	2,293
Colon_Xenograft	27,121	164,944	993	156,630	148,677	140,000	2,847
Colon Primary	14,067	164,944	5,164	140,191	123,818	100,704	395
Ovarian_Blood	26,956	164,944	1,150	156,224	147,600	138,270	1,760
Ovarian_Xenograft	26,617	164,944	731	158,306	151,434	142,258	7,020
Sarcoma_Blood	25,172	164,944	2,444	149,385	135,569	122,575	9,459
Sarcoma_Xenograft	24,235	164,944	1,831	153,998	144,057	131,133	6,275

TableS4b: Amplicons Coverage Statistics for the samples sequenced

Sample	mean coverage	number of amplicons with coverage:					
		>=0	=0x	>1000x	>5000x	>10,000x	>50,000x
CAL-A	24,914	676	-	668	648	607	-
CAL-B	26,059	676	1	659	618	571	4
CAL-C replicate 1	23,843	676	-	664	622	588	-
CAL-C replicate 2	23,488	676	-	667	634	588	1
CAL-D	26,617	676	-	661	618	565	3
CAL-B WGA1	22,674	676	-	670	649	609	5
CAL-B WGA2	20,548	676	-	670	648	606	3
Breast_Blood	25,044	676	1	655	623	580	2
Breast_Xenograft	24,005	676	1	665	627	583	-
Breast Xenograft WGA	17,396	676	1	667	624	536	2
Breast Primary	21,696	676	1	665	642	597	1
Colon_Blood	27,572	676	2	658	627	591	2
Colon_Xenograft	27,121	676	1	661	632	594	10
Colon Primary	14,067	676	1	664	622	511	-
Ovarian_Blood	26,956	676	1	661	632	592	4
Ovarian_Xenograft	26,617	676	1	667	639	594	25
Sarcoma_Blood	25,172	676	2	644	598	527	12
Sarcoma_Xenograft	24,235	676	2	656	616	567	19

TableS5a: Number of mutations detected (True Positive/False Positive) using all calibration samples both as training (rows) and tested samples (columns)

		Tested samples				
		**CAL-A**	**CAL-B**	**CAL-C1**	**CAL-D**	**CAL-C2**
	**Expected**	**196/0**	**200/0**	**201/0**	**201/0**	**201/0**
**Training samples**	**CAL-A**	175/6	163/3	181/3	181/4	177/1
	**CAL-B**	174/8	168/3	181/2	188/4	181/1
	**CAL-C1**	174/8	168/3	183/2	186/4	182/1
	**CAL-D**	170/6	164/2	179/1	187/2	180/1
	**CAL-C2**	179/12	175/11	184/10	188/12	185/2

TableS5b: Performance metrics (Sensitivity/Positive Predictive Value) using all calibration samples both as training (rows) and tested samples (columns)

		Tested samples				
		**CAL-A**	**CAL-B**	**CAL-C1**	**CAL-D**	**CAL-C2**
**Training samples**	**CAL-A**	89.3/96.7	81.5/98.2	90/98.4	90/97.8	88.1/99.4
	**CAL-B**	88.8/95.6	84/98.2	90/98.9	93.5/97.9	90/99.5
	**CAL-C1**	88.8/95.6	84/98.2	91/98.9	92.5/97.9	90.5/99.5
	**CAL-D**	86.7/96.6	82/98.8	89.1/99.4	93/98.9	89.6/99.4
	**CAL-C2**	91.3/93.7	87.5/94.1	91.5/94.8	93.5/94	92/98.9

Table S6 : Average coverage of amplicons after random sampling of reads for 3 calibration samples

Sample	Simulated Multiplex Level	Average Amplicon Coverage
CAL-B	2	13,026.1
	4	6,514.2
	8	3,256.0
	16	1,630.2
	32	814.7
CAL-C	2	11,922.8
	4	5,959.3
	8	2,979.9
	16	1,490.0
	32	744.1
CAL-D	2	13,310.9
	4	6,655.5
	8	3,326.2
	16	1,662.7
	32	830.6

TableS7: Filtering of low prevalence mutations in the cancer camples

	Breast				Colon			Ovarian		Sarcoma	
	**germline**	**Xenograft**	**Xenograft+ WGA**	**Primary**	**germline**	**Xenograft**	**Primary**	**germline**	**Xenograft**	**germline**	**Xenograft**
Training Sample	CAL-A	CAL-B	CAL-C2	CAL-C2	CAL-A	CAL-A	CAL-C2	CAL-A	CAL-A	CAL-B	CAL-B
No. of mutations called ≥5% prevalence	37	62	57	42	37	50	47	33	35	37	44
novel (not in dbSNP)	*1*	*23*	*21*	*2*	*0*	*12*	*10*	*1*	*1*	*1*	*13*
Raw somatic mutations	NA	16	16	1	NA	11	10	NA	2	NA	12
With Mouse Evidence		*15*	*15*	*0*		*0*	*0*		*1*		*12*
Filtered somatic mutations		1	1	1		11	10		1		0

NA: not applicable

TableS8: List and annotation of the somatic mutations identified by UDT-Seq in the cancer samples

Tumor site	Sample	Mutation Type	Chr	hg19 coordinate	Ref	Alt	Prevalence	Gene	Transcript	amino-acid substitution
Colon	Primary	misense	chr4	1,806,599	C	T	0.47	FGFR3	NM_022965	R327C
			chr5	149,439,266	C	T	0.43	CSF1R	NM_005211	R710H
			chr5	112,128,185	C	T	0.40	APC	NM_000038	R230C
			chr12	25,398,284	C	T	0.35	KRAS	NM_033360	G12D
			chr17	29,588,809	C	A	0.31	NF1	NM_001042492	P1553H
			chr4	55,561,755	C	T	0.10	KIT	NM_000222	R49C
		nonsense	chr5	112,154,942	C	T	0.42	APC	NM_000038	R405X
			chr5	112,151,204	C	T	0.23	APC	NM_000038	R283X
		intronic	chr17	7,573,888	G	A	0.35	TP53	NA	
			chr7	140,481,498	A	G	0.10	BRAF	NA	
	Xenograft	misense	chr19	1,221,995	C	T	0.66	STK11	NM_000455	R304W
			chr4	1,806,599	C	T	0.59	FGFR3	NM_022965	R327C
			chr5	149,439,266	C	T	0.56	CSF1R	NM_005211	R710H
			chr11	533,839	G	A	0.55	HRAS	NM_005343	R73C
			chr12	25,398,284	C	T	0.51	KRAS	NM_033360	G12D
			chr17	29,588,809	C	A	0.51	NF1	NM_001042492	P1553H
			chr5	112,128,185	C	T	0.49	APC	NM_000038	R230C
			chr8	38,274,845	G	C	0.44	FGFR1	NM_023106	L457V
		nonsense	chr5	112,154,942	C	T	0.51	APC	NM_000038	R405X
			chr5	112,151,204	C	T	0.49	APC	NM_000038	R283X
		intronic	chr17	7,573,888	G	A	0.47	TP53	NA	
Breast	Primary	misense	chr11	534,288	C	A	0.51	HRAS	NM_005343	G12V
	Xenograft	misense	chr11	534,288	C	A	0.48	HRAS	NM_005343	G12V
	Xenograft+WGA	misense	chr11	534,288	C	A	0.48	HRAS	NM_005343	G12V
Ovarian	Xenograft	misense	chr17	7,577,538	C	T	1.00	TP53	NM_001126113	R248Q

NA: Not applicable

Table S9:Validation by independent assay of 11 mutations identified in the colon cancer samples.

	Observed Prevalence			
	UDT-Seq		validation assay (Sanger or SNaPshot)	
**Mutation**	**Xenograft**	**Primary**	**Xenograft**	**Primary**
APC-R283X	0.51	0.23	0.50	0.25
APC-R405X	0.49	0.42	0.50	0.43
CSF1R-R710H	0.56	0.43	0.40 [3]	0.40 [3]
FGFR1-L538V	0.44	<0.05 [2]	0.20	ND
FGFR3-R327C	0.59	0.47	0.50 [3]	0.40 [3]
HRAS-R73C	0.55	<0.05 [2]	0.50	ND
KIT-R49C	<0.05 [2]	0.10	ND [3]	0.10 [3]
KRAS-G12D	0.51	0.35	0.50	0.38
NF1-P1553H	0.51	0.31	0.70	0.50
STK11-R304W	0.66	0.23 [1]	0.76	0.55
TP53-intron	0.47	0.35	0.40	0.40

1 estimated from visual inspection

2. below UDT-Seq detection threshold of 5% prevalence

3. by Sanger Sequencing

ND not detected

Table S10: Primers and probes used in the validation assays

Mutation	Forward(5'->3')	Reverse(5'->3')	SNaPshot probe
APC-R283X	GACACTTCATTTGGAGTACCTTAACA	GGCATTAGTGACCAGGGTTT	C(3)GTGCTTAATTTTTAGGGTTCAACTACA
APC-R405X	TATCCATGCGACAGTCTGGA	CCACTCCCAACAGGTTTCAC	C(11)AGAGAGGCAGGCGTGAAATC
CSF1R-R710H	ATAGCCACCCATTCATGAGC	CTATGGCGACCTGCTCAACT	Sanger
FGFR1-L538V	TAACCCCCTTCCCTAGCTGT	TTCTTTAAAGCGGACGCAAC	C(15)ATCCTGCGTGCAGGCCCCCA
FGFR3-R327C	TTCATTCAATGCTGGTGGAA	CTCGAGCTCGGAGACATTG	Sanger
HRAS-R73C	TGGTGTTGTTGATGGCAAAC	AGAGGCTGGCTGTGTGAACT	AAAACACACACAGGAAGCCCTCCCCGGTGC
KIT-R49C	TGTTTTTCTTGGCAGGCTCT	GTTGGTGCACGTGTATTTGC	Sanger
KRAS-G12D	TATCGTCAAGGCACTCTTGC	CGTCTGCAGTCAACTGGAAT	C(21)AAGGCACTCTTGCCTACGCCA
NF1-P1553H	GCTGTTGGAAGACGACCTTT	TGGTGGCAAACTCTCCTTCT	ACCTGGGTCCTCCAGAGCACAAAC
STK11-R304W	GGGATGCTTGAGTACGAACC	AACAGGACACTGCCCAGAGA	CAAGCCAAGAGGTTCTCCATCCGGCAGATC
TP53-intron	GAAGGCAGGATGAGAATGGA	ACTTCTCCCCCTCCTCTGTT	C(16)ATGGCTTTCCAACCTAGGAAG
